# PLAG1 and USF2 Co-regulate Expression of Musashi-2 in Human Hematopoietic Stem and Progenitor Cells

**DOI:** 10.1016/j.stemcr.2018.03.006

**Published:** 2018-04-10

**Authors:** Muluken S. Belew, Sonam Bhatia, Ava Keyvani Chahi, Stefan Rentas, Jonathan S. Draper, Kristin J. Hope

**Affiliations:** 1 Department of Biochemistry and Biomedical Sciences, Stem Cell and Cancer Research Institute, McMaster University, 1280 Main Street West, Hamilton, ON L8S 4K1, Canada

**Keywords:** human hematopoietic stem cells, self-renewal, promoter, transcriptional regulation, transcription factors, Musashi-2, genome-wide DNA binding site mapping, PLAG1, USF2

## Abstract

MSI2, which is expressed predominantly in hematopoietic stem and progenitor cells (HSPCs), enforces HSPC expansion when overexpressed and is upregulated in myeloid leukemias, indicating its regulated transcription is critical to balanced self-renewal and leukemia restraint. Despite this, little is understood of the factors that enforce appropriate physiological levels of MSI2 in the blood system. Here, we define a promoter region that reports on endogenous expression of *MSI2* and identify USF2 and PLAG1 as transcription factors whose promoter binding drives reporter activity. We show that these factors co-regulate, and are required for, efficient transactivation of endogenous *MSI2*. Coincident overexpression of USF2 and PLAG1 in primitive cord blood cells enhanced *MSI2* transcription and yielded cellular phenotypes, including expansion of CD34+ cells *in vitro*, consistent with that achieved by direct MSI2 overexpression. Global chromatin immunoprecipitation sequencing analyses confirm a preferential co-binding of PLAG1 and USF2 at the promoter of *MSI2*, as well as regulatory regions corresponding to genes with roles in HSPC homeostasis. PLAG1 and USF2 cooperation is thus an important contributor to stem cell-specific expression of MSI2 and HSPC-specific transcriptional circuitry.

## Highlights

• We define a regulatory region governing physiological MSI2 expression in human HSPCs
• USF2 and PLAG1 collaboratively control endogenous HSPC-specific MSI2 expression
• MSI2 expression and stemness is maintained in culture upon USF2 and PLAG1 co-overexpression
• USF2 and PLAG1 exhibit genomic co-localization and associate with autophagy genes

## Teaser

MSI2 is an essential human hematopoietic stem and progenitor cell (HSPC) regulator, but knowledge of the mechanisms ensuring its appropriate expression in this context are lacking. Here, Hope and colleagues map the MSI2 promoter functional in hematopoietic cells and identify USF2 and PLAG1 as essential, cooperative enforcers of endogenous MSI2 expression and stemness traits in human HSPCs.

## Introduction

The unique ability of hematopoietic stem cells (HSCs) to self-renew while giving rise to mature cells by differentiation enables their regenerative capacity. As such, identification and characterization of key regulators of this property are of both fundamental and clinical interest. The Musashi-2 (MSI2) RNA-binding protein is now recognized as one such key HSC regulator. Its expression is highest in the primitive HSC compartment and progressively decreases upon commitment (de Andres-Aguayo et al., 2011, Hope et al., 2010, Park et al., 2014, Rentas et al., 2016). MSI2 loss of function results in a significant depletion in the reconstitution capacity of primitive murine hematopoietic cells (de Andres-Aguayo et al., 2011, Hope et al., 2010, Ito et al., 2010, Park et al., 2014). By contrast, when moderately overexpressed, it imparts enhanced HSC self-renewal activity as measured by increased competitiveness in *in vivo* reconstitution assays (Hope et al., 2010). In the human system we have shown an analogous detrimental effect on cord blood (CB) HSC-mediated reconstitution when MSI2 is repressed. These same stem cells undergo significant *ex vivo* expansion when MSI2 is overexpressed (Rentas et al., 2016). MSI2 has also been implicated in aspects of leukemia pathogenesis (Kharas et al., 2010, Park et al., 2015, Ito et al., 2010). For instance, in mouse models of chronic myeloid leukemia (CML) and myelodysplastic syndrome (MDS), ectopic expression of MSI2 encourages promotion of the disease to acute phases (Kharas et al., 2010, Taggart et al., 2016). In the human context, aberrantly high expression of MSI2 correlates with more aggressive CML disease states and is associated with poor prognosis in acute myeloid leukemia and MDS (Ito et al., 2010, Kharas et al., 2010, Taggart et al., 2016). Taken together, these studies suggest that the precise molecular regulation of MSI2 gene expression may be among the critical mechanisms underlying balanced HSC self-renewal/differentiation and the restraint of leukemia progression. Despite the importance of MSI2 in stem cell behavior, it remains poorly understood how *MSI2* expression is maintained at appropriate levels, and very little is known of the promoter elements or transcription factors (TFs) that mediate this. Here, we report an approach to address HSC cell fate control through the systematic dissection of the *MSI2* promoter functional in hematopoietic cells. Through this strategy, we have identified two TFs that function as cooperative regulators of *MSI2* and that together play a key role in HSPC function.

## Results

### Dissection of the *MSI2* Minimal Promoter

**Figure 1:**
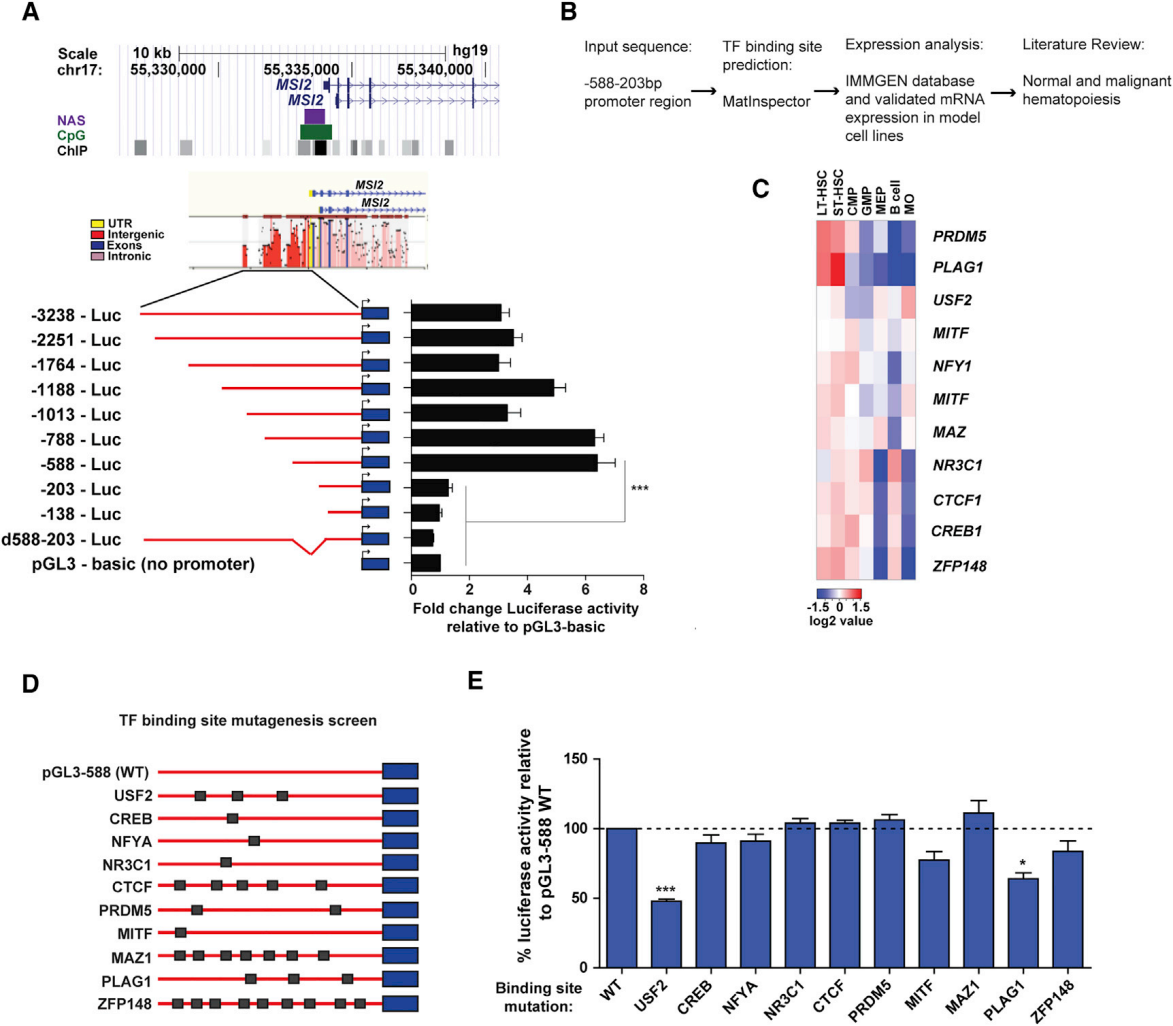
*In Silico* Mapping and Mutagenesis Screening Identifies the *MSI2* Promoter in Hematopoietic Cells with Dependence on USF2 and PLAG1 Binding Sites for Activity (A) UCSC genome browser annotation of features within the region directly 5′ upstream of *MSI2* (top panel) including ChIP-validated transcription factor (TF) binding sites, a CpG island, and nuclease accessible site (NAS). Middle panel depicts *MSI2* genomic sequence alignment and homology between mouse and human species as analyzed by MULAN. Bottom panel shows a schematic representation of the serial 5′- promoter truncations (red) placed upstream of the firefly luciferase (Luc) reporter gene (blue) and their corresponding luciferase reporter activity. (B) Workflow of TF selections for binding site mutagenesis screen. (C) Heatmap demonstrating the relative expression across the hematopoietic hierarchy of a prioritized subset of TFs predicted *in silico* to bind the *MSI2* promoter. (D) Schematic depicting the binding sites mutated for each of the ten candidate *MSI2*-regulating TFs in independent reporter constructs. (E) Percentage changes in luciferase activity after specific TF binding site mutagenesis within the minimal promoter sequence compared with the wild-type (WT) promoter. Data for all reporter assays was generated from n = 3–6 independent experiments. Data are presented as means ± SEM. ^∗^p < 0.05, ^∗∗∗^p < 0.001. See also Tables S1 and S2.

MSI2 expression is evolutionarily conserved in both mouse and human HSPCs. Therefore, as an initial step in mapping its promoter we concentrated on the region directly upstream of the translational start site sharing extensive sequence similarity between the two species. This corresponded to a region extending to ∼3.2 kb upstream wherein homology peaks were detected throughout as identified by the multiple sequence local alignment and visualization tool (MULAN) (Ovcharenko et al., 2004) (Figure 1A, middle panel). Multiple sequence features including a nuclease accessible site (NAS), CpG island, and TF binding sites as identified by chromatin immunoprecipitation sequencing (ChIP-seq) within a conserved region ∼1 kb upstream of the translational start site further suggested the potential for this region to function in a promoter capacity (Figure 1A). Introduction of this 3.2 kb region upstream of firefly luciferase in pGL3-basic yielded significantly greater reporter activity compared with the promoterless construct in MSI2-expressing K562 and HEK293 cell lines (3-fold and 7.5-fold respectively) (Figure 1A, data not shown). Using variations in the extent of homology peaks as endpoints, we generated a set of luciferase reporter constructs with serial 5′-truncations of the ∼3.2 kb sequence. A significant drop in reporter activity resulted only when the upstream sequence driving reporter expression was reduced from −588 to −203 bp (Figure 1A). In confirmation that a minimal promoter region containing essential *cis* elements governing *MSI2* expression is contained within this 385 bp region we found its deletion from the full-length 3.2 kb fragment was sufficient to repress luciferase activity to the level of the promoterless reporter (Figure 1A).

### USF2 and PLAG1 Binding Sites Are Required for *MSI2* Promoter Activity

We next implemented a mutagenesis screen to systematically test the functionality of TF consensus sites within the minimal promoter region in order to pinpoint key *MSI2*-regulators. First we used *in silico* TF binding site prediction (MatInspector) to identify a total of 107 TF candidates representing 65 different TF families. From within this set we removed those TFs not appreciably expressed in both murine and human HSPCs, where *MSI2* expression is known to be concentrated. A final refinement based on an extensive literature search to identify novel TFs, or those implicated in the regulation of stem cell function and/or leukemia, yielded a list of ten candidate regulators of primitive hematopoietic cell expression of *MSI2* (Figures 1B and 1C). Using the −588-Luc reporter construct as a template we generated a series of constructs possessing mutations of the consensus binding sites of each of these TFs and screened for loss of luciferase reporter activity (Figures 1D and 1E). The only TFs whose binding site mutagenesis resulted in a significant reduction in reporter activity were the E-box motif-binding USF2 and the C_2_H_2_ zinc finger protein PLAG1 (Figure 1E). The results of our rationalized screen thus indicate that intact PLAG1 and USF2 binding sites are required for full *MSI2* promoter activity.

### USF2 and PLAG1 Bind the Promoter of *MSI2* and Promote Its Transcription

**Figure 2:**
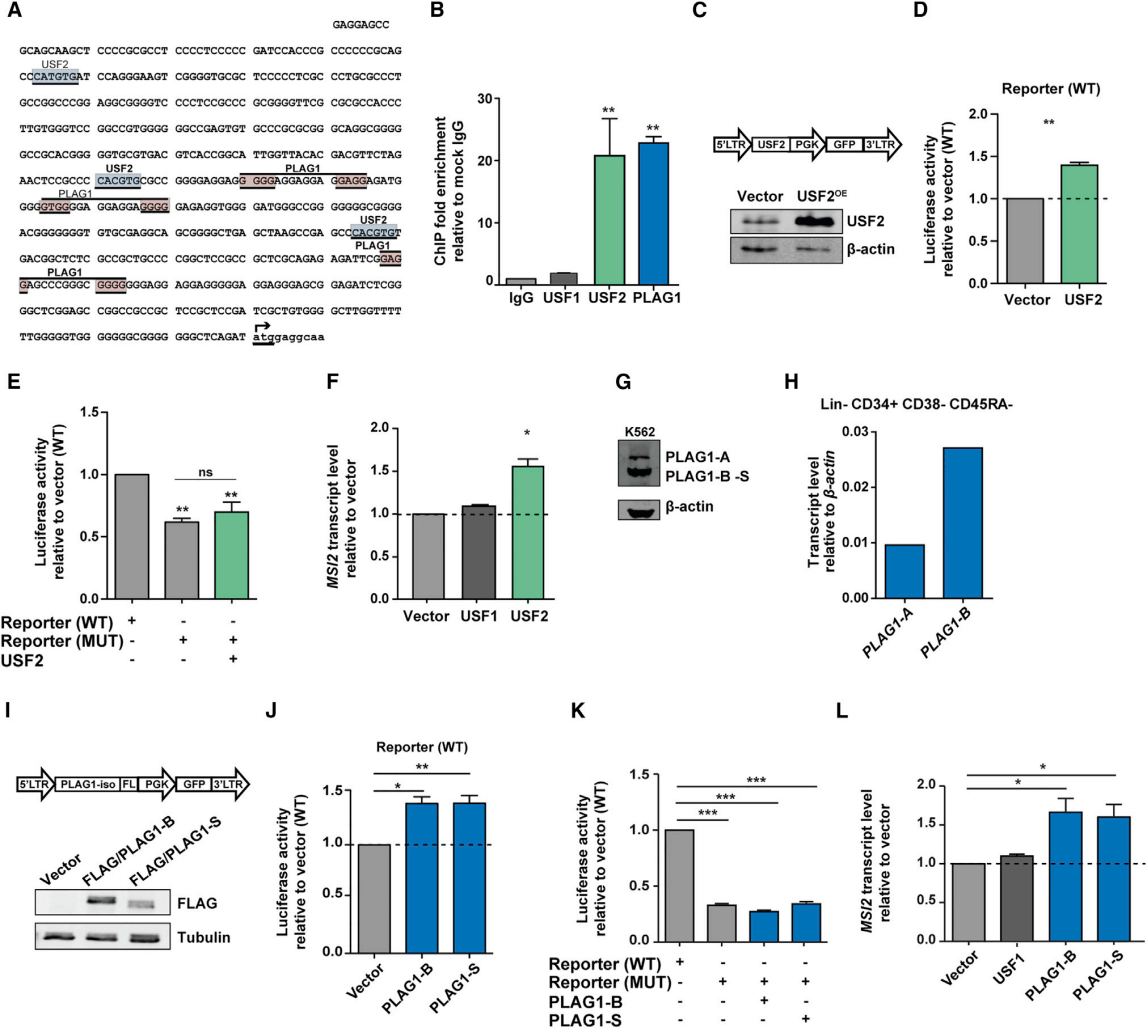
Ectopic PLAG1 and USF2 Bind the *MSI2* Promoter and Enhance Its Transcription in K562 Cells (A) DNA sequence of the minimal promoter for *MSI2* with predicted (MatInspector) USF2 (blue) and PLAG1 (red) binding sites shown. The translation start site of *MSI2* is identified by the forward arrow from lower case nucleotides and the initiation codon is underlined. (B) ChIP-qPCR for PLAG1 and USF2 binding of the minimal *MSI2* promoter (n = 4 experiments). (C) USF2-overexpressing lentiviral construct schematic (top) and western blot quantification of its enhanced protein levels in K562 cells (bottom). (D) Wild-type (WT) *MSI2* minimal promoter reporter construct luciferase activity upon USF2 overexpression (n = 3 experiments). (E) Luciferase activity of USF2 binding site-mutated (MUT) reporter with and without USF2 overexpression compared with WT (n = 4 experiments). (F) Endogenous *MSI2* transcript levels following USF2 overexpression in K562 cells (n = 3 experiments). (G) Characterization of PLAG1 isoform expression by western blot in K562 cells. (H) Comparison of PLAG1-A and PLAG1-B expression in flow-sorted CB HSPCs (performed once from pooled CB samples). (I) FLAG-tagged PLAG1 short-isoform overexpressing lentiviral construct schematic and western blot quantification of enhanced protein levels in K562 cells (bottom). (J) WT reporter construct luciferase activity upon PLAG1-B or PLAG1-S overexpression (n = 4 experiments). (K) Luciferase activity of PLAG1-B and PLAG1-S MUT reporters with PLAG1-B or PLAG1-S overexpression compared with WT (n = 4 experiments). (L) Endogenous *MSI2* transcript levels following PLAG1-B or PLAG1-S overexpression in K562 cells (n = 3 experiments). Data are presented as means ± SEM. ^∗^p < 0.05, ^∗∗^p < 0.01, ^∗∗∗^p < 0.001. See also Figure S1 and Table S3.

The minimal *MSI2* promoter sequence contains a total of three consensus E-box motifs for USF2 (Bendall and Molloy, 1994) and three G-rich bipartite consensus sequences for PLAG1 (Hensen et al., 2002) (Figure 2A). In support of the functionality of these binding sites, ChIP-qPCR demonstrated that PLAG1 and USF2 yield significant fold enrichment at the minimal promoter region of *MSI2* in K562 cells (Figure 2B). Combined with the mutagenesis data, these results are consistent with the direct binding of USF2 and PLAG1 to their consensus recognition elements as requirements for full activity of the *MSI2* minimal promoter. To quantify the transactivation potential of USF2 and/or PLAG1 on the promoter region we independently overexpressed each gene. USF2-overexpressing (USF2) cells exhibited a significant 40% (1.4-fold) enhanced reporter activity compared with vector controls (Figures 2C and 2D). In addition, USF2-overexpressing cells did not exhibit any elevation of luciferase reporter levels when transfected with a promoter construct that had mutations in all three USF2 binding sites, further confirming the specificity of USF2 binding to the predicted sites (Figure 2E). Lastly, USF2 overexpression resulted in a 1.4-fold increased *MSI2* transcript level compared with negative controls. Binding to the E-box motif within the *MSI2* promoter and transactivation of *MSI2* expression is specific to USF2 as its closely related family member, USF1, does not bind the *MSI2* promoter (Figure 2B) and yielded no increase in *MSI2* expression when overexpressed (Figure 2F). Together, these results validate the reporter results and confirm the direct connection of USF2 levels with endogenous *MSI2* expression.

There are three human PLAG1 transcript variants encoding two protein isoforms. The larger isoform (PLAG1-A) contains all annotated functional domains, whereas the shorter form (PLAG1-B) is missing Zn fingers F1 and F2. A third reported slightly smaller PLAG1 translational isoform exists through translation from methionine 100 (PLAG1-S), is missing the first 17 N-terminal residues of PLAG1-B, and thus co-migrates with it on SDS-PAGE (Debiec-Rychter et al., 2001) (Figures 2G and S1). In both K562 and primary primitive human CB cells, PLAG1 appears to be contributed largely by the short-form(s) (Figures 2G and 2H). Thus to characterize their respective contributions to *MSI2* transactivation we overexpressed PLAG1-B and PLAG1-S or the negative control USF1 in K562 cells (Figure 2I). Both PLAG1 forms independently enhanced reporter activity ∼1.4 fold in a PLAG1-binding-site-dependent manner (Figures 2J and 2K) and, most importantly, resulted in a significant 1.6-fold increase in endogenous *MSI2* transcription, suggesting they behave interchangeably with respect to *MSI2* regulation (Figure 2L). Together, these findings indicate that both USF2 and PLAG1 directly interact with the *MSI2* minimal promoter and are capable of ectopically transactivating its expression.

**Figure 3:**
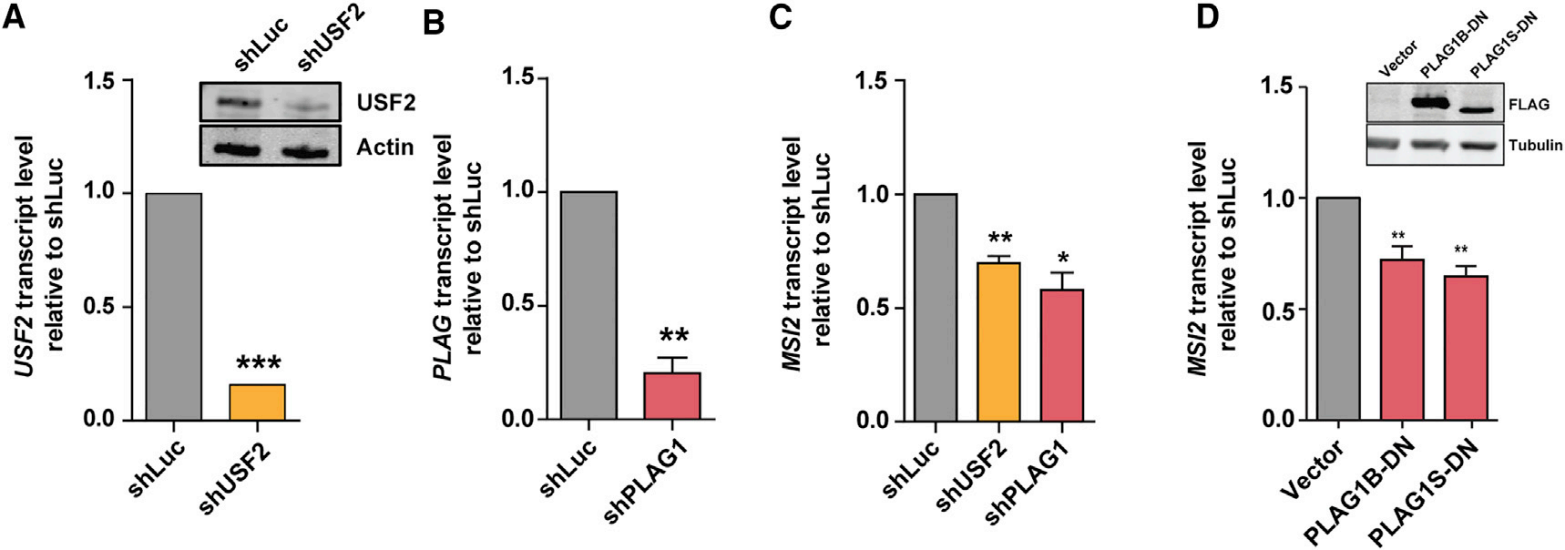
PLAG1 and USF2 Are Required for *MSI2* Promoter Activity and Expression (A and B). Knockdown efficiencies of shUSF2 (E) and shPLAG1 (F) as measured post-infection by q-RT-PCR and western blot where sufficient cells were available (inset, A) in K562 cells (n = 3 experiments for each hairpin). (C) *MSI2* transcript level reductions following *USF2* and *PLAG1* knockdown (n = 3 experiments). (D) Western blot (inset) validation of overexpression of the dominant-negative forms of PLAG1-B (PLAG1B-DN) and PLAG1-S (PLAG1S-DN), and graph of *MSI2* transcript levels following introduction of PLAG1B-DN or PLAG1S-DN into K562 cells. (n = 3 experiments). Data are presented as means ± SEM. ^∗^p < 0.05, ^∗∗^p < 0.01, ^∗∗∗^p < 0.001. See also Table S3.

Reciprocal knockdown experiments with short hairpin RNAs (shRNAs) targeting *USF2* and *PLAG1* resulted in a 40% and 50% decrease in endogenous *MSI2* transcription, respectively, in comparison with control cells expressing non-targeting shRNA (shLuc) (Figures 3A–3C). In addition, we measured the effects of PLAG1 loss of function using a dominant-negative approach wherein we introduced short PLAG1 forms missing their transactivation domains (Kas et al., 1998, Voz et al., 2000) (PLAG1-B DN and PLAG1-S-DN, respectively) (Figure 3D). The introduction of either form led to the attenuation of *MSI2* transcription by approximately 1.4-fold compared with controls (Figure 3D). These results confirm that PLAG1 transactivation activity is a requirement for maintenance of physiological levels of *MSI2* transcription. In summary, these gain- and loss-of-function assays demonstrate that USF2 and PLAG1 constitute critical components of the regulatory circuitry controlling *MSI2* transcription and are essential for maintaining physiological MSI2 expression levels.

### USF2 and PLAG1 Function Collaboratively to Promote *MSI2* Transactivation

**Figure 4:**
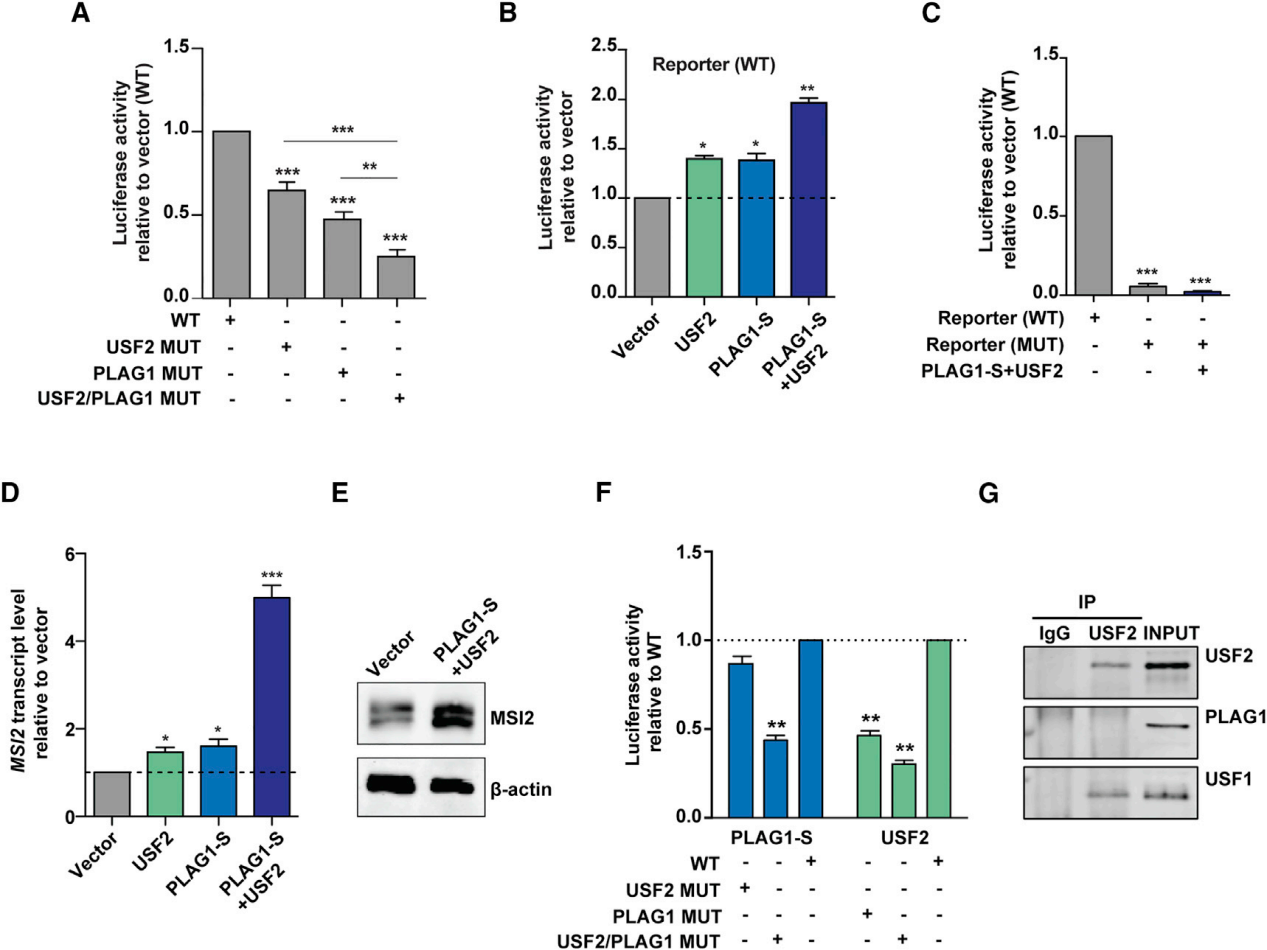
PLAG1-S and USF2 Collaboratively Regulate *MSI2* Promoter Activity and *MSI2* Expression (A) Reporter activity upon combinatorial binding site mutagenesis for USF2 and PLAG1 within the *MSI2* minimal promoter region. n = 11 independent experiments. (B) Reporter construct luciferase activity upon independent or coincident overexpression of USF2 and PLAG1-S (n = 3 experiments). (C) Luciferase activity of PLAG1 and USF2 MUT reporters upon PLAG1-S and USF2 co-overexpression (n = 3 experiments). (D and E) Endogenous *MSI2* transcript (D) (n = 3 experiments) and protein levels (E) following overexpression of PLAG1-S and USF2 in K562 cells compared with vector control. (F) Luciferase reporter activity of the *MSI2* minimal promoter region upon PLAG1-S or USF2 overexpression when the binding site for the reciprocal factor or for both factors is mutated (MUT) (n = 3 experiments). (G) Co-immunoprecipitation analysis performed on K562 cells using anti-USF2 for immunoprecipitation and immunoblotting with antibodies to USF2, the known USF2 interacting partner USF1 or PLAG1. Data are presented as means ± SEM. ^∗^p < 0.05, ^∗∗^p < 0.01, ^∗∗∗^p < 0.001. See also Table S3.

We next tested whether Plag1 and USF2 cooperate to transactivate *MSI2* expression. First we generated a minimal promoter reporter assay containing mutations in both the PLAG1 and USF2 consensus sites. Combinatorial binding site mutagenesis yielded a more significant reduction in minimal promoter activity than when PLAG1 or USF2 binding sites were mutated in isolation (Figure 4A). As this suggested that USF2 and PLAG1 potentially cooperate to regulate *MSI2* promoter activity we sought to further address this possibility using PLAG1-S and USF2 co-overexpression in K562 cells. Luciferase activity following co-overexpression was approximately 2-fold greater than in negative controls and over 1.5-fold greater than in cells overexpressing either USF2 or PLAG1-S alone (Figure 4B). In addition, the same assay performed in singly and doubly USF2- and PLAG1-S-overexpressing cells transfected with promoter reporter constructs with co-mutated USF2/PLAG1 binding sites demonstrated background level reporter activity, confirming the specificity of the predicted binding sites (data not shown and Figure 4C). Strikingly, co-overexpression increased endogenous *MSI2* transcription more than 4.5-fold in comparison with vector transfected control cells, an increase that was also manifested at the protein level (Figures 4D and 4E). This robust increase in *MSI2* transcription is more than 2.5-fold higher than that observed in cells overexpressing USF2 or PLAG1-S independently, indicating a potential synergistic response on wholesale *MSI2* transactivation when the two factors are coordinately elevated. To assess the nature of their collaboration on the minimal promoter of *MSI2* we performed reporter assays in single factor overexpressing cells carrying the promoter sequence containing mutations of the alternate factor's consensus sites. We find that when USF2 binding sites are mutated, transactivation by PLAG1 is unaffected, whereas when PLAG1 binding sites are mutated transactivation of the promoter by USF2 is attenuated by more than 2-fold (Figure 4F). This is intriguingly suggestive of a requisite role for PLAG1 in the recruitment of cooperative TFs. To further characterize the potential cooperation between PLAG1 and USF2 we used co-immunoprecipitation (coIP) to test whether the two TFs are involved in a protein-protein interaction and found that they are not engaged in an interaction detectable by coIP (Figure 4G). Thus, although PLAG1 and USF2 exhibit an epistatic relationship, these data suggest that PLAG1 and USF2 do so in the absence of forming a direct, stable physical interaction.

**Figure 5:**
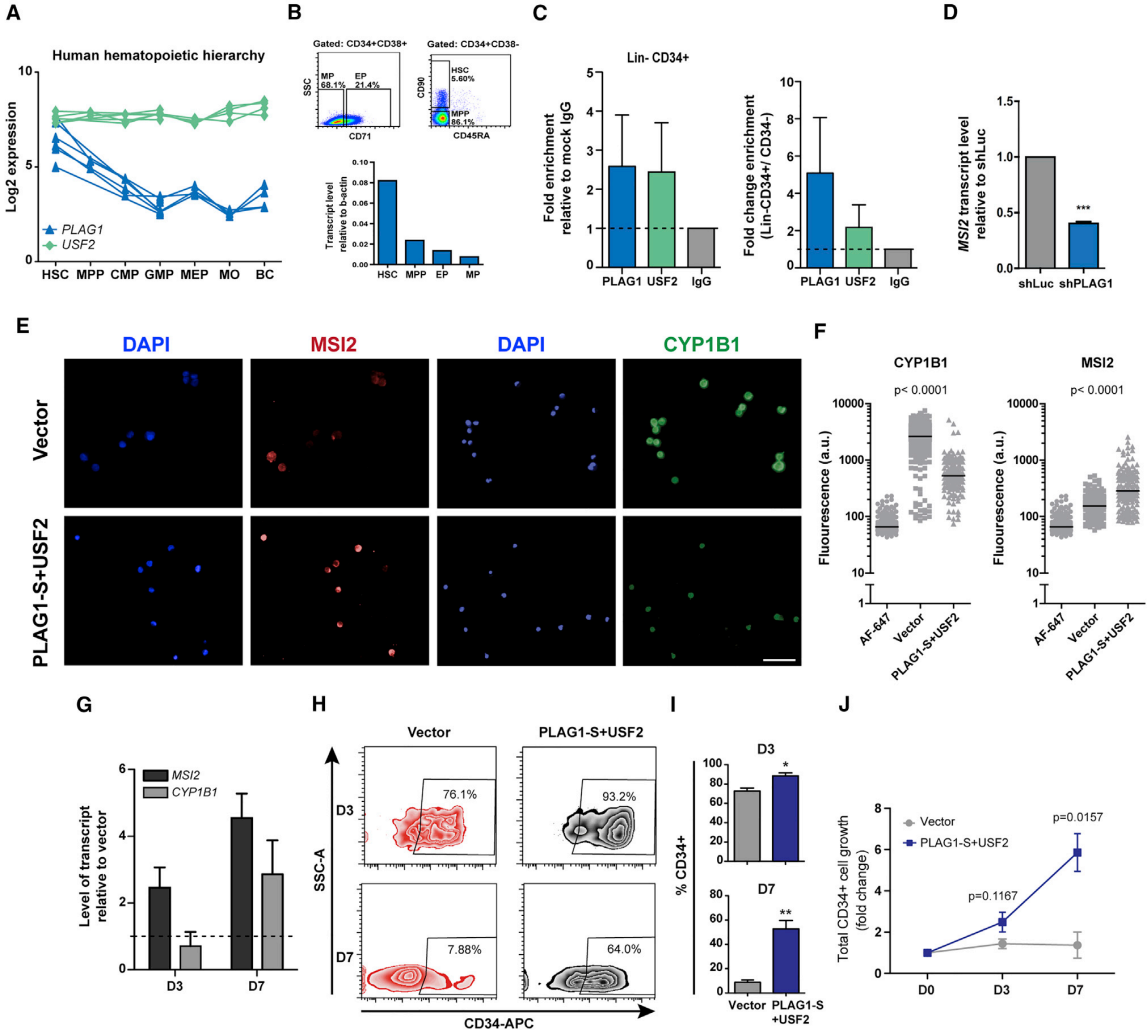
PLAG1-S and USF2 Co-regulate *MSI2* Expression and Its Downstream Functions in Primitive Hematopoietic Cells (A) Expression profile of PLAG1 and USF2 in the human hematopoietic hierarchy. BC, band cell; CMP, common myeloid progenitor; GMP, granulocyte monocyte progenitor; MEP, megakaryocyte erythroid progenitor; MO, monocytes; MPP, multipotent progenitors. (B) Flow cytometry gating strategy to isolate erythroid progenitors (EP), myeloid progenitors (MP), MPP and HSCs from cord blood (top) and the q-RT-PCR validated expression of PLAG1-B within these populations (bottom, performed once from pooled CB samples). (C) ChIP-qPCR-defined fold enrichment of PLAG1 and USF2 at the *MSI2* promoter in purified cord blood Lin-CD34+ (left) and fold change ChIP enrichment in Lin-CD34+ compared with Lin-CD34- (right) (n = 3 experiments). (D) qPCR determination of *MSI2* transcript levels following *PLAG1* knockdown in Lin-CD34+ cells (n = 3 experiments). (E) Representative immunofluorescence images of MSI2 and CYP1B1 proteins in Lin-CD34+ CB cells expressing control vector or co-overexpressing PLAG1-S and USF2 and cultured for 7 days. Scale bar, 25 μm. (F) Quantification of MSI2 and CYP1B1 expression on a per cell basis. Each point in the graph represents an individual stained cell and the solid line denotes the mean fluorescence intensity of each dataset. Cells stained with Alexa Fluor 647 (AF-647) conjugated secondary antibody alone were considered as background fluorescence controls (>100 cells analyzed per condition for each experiment, plots summarize n = 3 experiments). (G) Fold change in the transcript level of *MSI2* and *CYP1B1* upon co-expression of PLAG1-S and USF2 at day 3 (D3) and day 7 (D7) of *in vitro* culture relative to vector control. (H) Representative flow cytometry plots showing the frequency of CD34+ cells at the D3 and D7 *in vitro* culture points with the co-overexpression of PLAG1-S and USF2. (I) Quantification of CD34+ cell frequency (n = 3 experiments). (J) Fold change in total CD34+ cells over the 7-day culture period. Data are presented as means ± SEM. ^∗^p < 0.05, ^∗∗^p < 0.01, ^∗∗∗^p < 0.001. See also Table S3.

If the cooperative effect of USF2 and PLAG1 is indeed a key component in driving *MSI2* expression in primary hematopoietic cells, it would be expected that maximal *MSI2* expression would be observed in cells where the combined levels of these two factors peak. Indeed, this is the case, as *USF2* is ubiquitously expressed across the human hematopoietic hierarchy, while *PLAG1* is at its highest levels in the most primitive HSC subset and declines rapidly in expression with differentiation (Figure 5A), a profile that is also consistent with *MSI2* expression (Rentas et al., 2016). Moreover, we confirmed PLAG1-B isoform expression follows the same pattern of being more highly expressed in HSC enriched CB populations (Figure 5B). To support that this maximal presence of PLAG1 in combination with USF2 in more primitive hematopoietic cells translates to an increase in actual *MSI2* promoter binding we performed ChIP to examine PLAG1 and USF2 binding to the −588 bp region of Lin-CD34+ HSPCs and Lin-CD34-restricted progenitors. ChIP-qPCR demonstrated that PLAG1 and USF2 do indeed directly bind to the *MSI2* promoter with more than 4.5- and 2.5-fold enrichment, respectively, in Lin-CD34+ compared with Lin-CD34-cells (Figure 5C). Furthermore, selective knockdown of PLAG1 in Lin-CD34+ cells results in a 60% down-regulation of *MSI2* transcript validating the PLAG1-dependent nature of MSI2 expression in primitive hematopoietic cells (Figure 5D).

### Co-overexpression of USF2 and PLAG1 Phenocopies Expression of Ectopic MSI2 and Enhances Primitive Cell Output

We have previously reported that MSI2 enforces HSPC expansion *ex vivo* in part through the post-transcriptional repression of the aryl hydrocarbon receptor (AHR) effector CYP1B1 (Rentas et al., 2016). As would be expected if a major effect of their cooperative function is MSI2 upregulation, co-ordinate overexpression of USF2 and PLAG1-S in Lin-CD34+ cells resulted not only in a significant 4.5-fold elevation in *MSI2* transcription at day 7 of *ex vivo* culture (D7) but also in the selective repression of CYP1B1 protein (and not its mRNA levels) (Figures 5E–5G). Most importantly, relative to controls, significant 5- and 6-fold enhancements in the proportion and total numbers of primitive CD34+ cells, respectively, were observed following a 7-day culture of CB cells jointly overexpressing USF2 and PLAG1-S (Figures 5H–5J). Together, these results are consistent with USF2 and PLAG1-S acting in combination to enforce MSI2 expression, thereby promoting primitive hematopoietic cell maintenance.

### Genome-wide Identification of USF2 and PLAG1 Co-occupancy Identifies HSC Regulators as a Subset of their Common Targets

**Figure 6:**
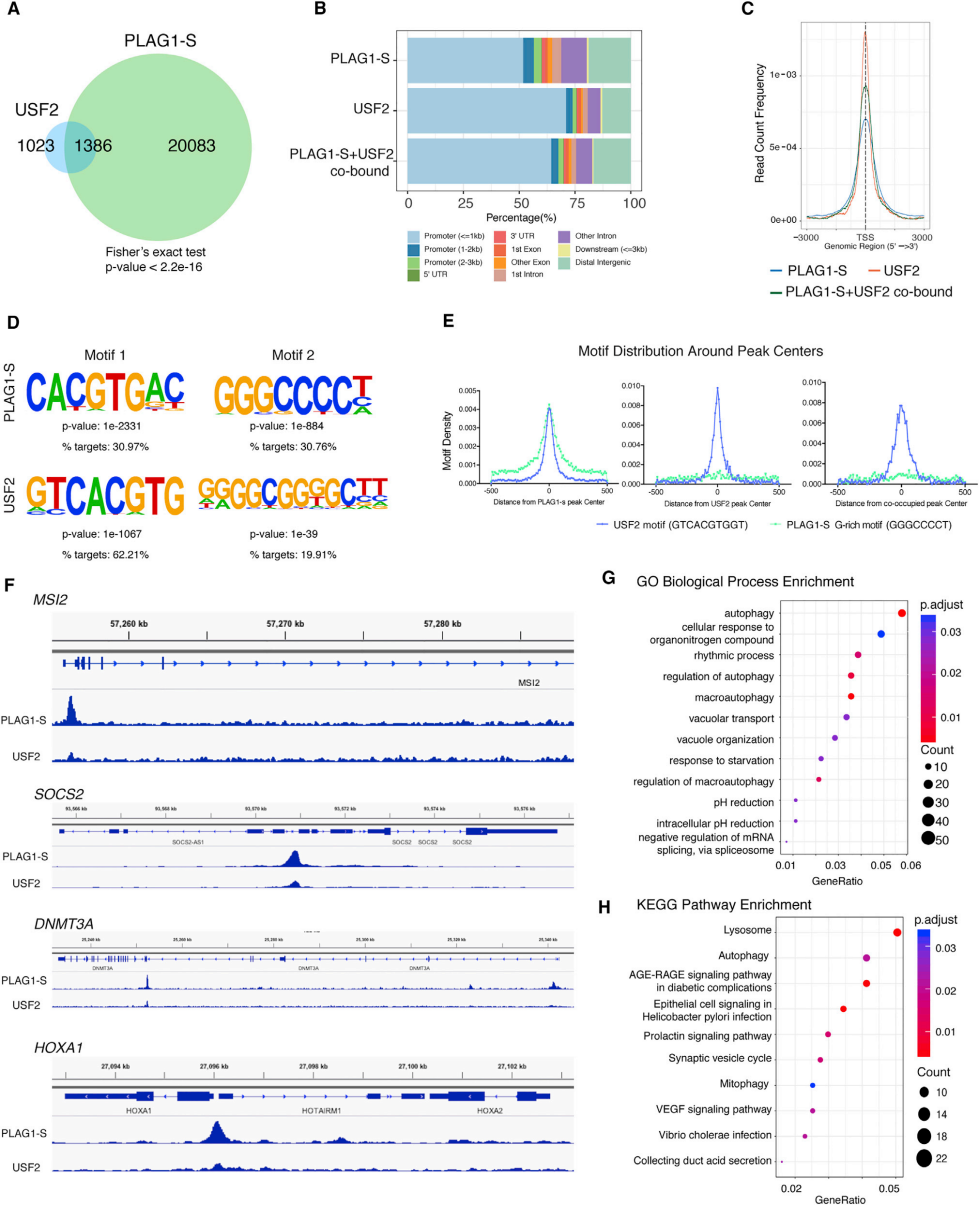
ChIP-Seq Mapping of USF2 and PLAG1-S Binding Identifies Co-occupancy at Promoters of *MSI2* and Other HSC Regulators (A) Venn diagram showing the overlap of USF2 and PLAG1-S ChIP peaks within a 100 bp distance. (B) Peak distribution profiles of all PLAG1-S and USF2 peaks, along with the co-occupied sites (PLAG1-S + USF2). (C) Read distribution profiles of peaks found within ±3 kb of the TSS. (D) *De novo* motif discovery analysis of the PLAG1-S and USF2 peaks. Top two most significantly enriched motifs identified by HOMER are shown for each factor. (E) Motif density distribution profiles around peak centers for the USF2 consensus and the G-rich motif identified as the PLAG1-S consensus (motif 2) during *de novo* motif discovery. (F) Integrative genome browser tracks showing the ChIP signal at regulatory regions of *MSI2*, *SOCS2*, *DNMT3A*, and *HOXA1*. (G and H) Enrichment plots showing the biological process (G) and KEGG (H) pathways enriched in PLAG1-S + USF2 co-occupied sites using ChIPseeker R package. Counts represent the number of genes identified in each cluster and p.adjust are the adjusted p values using BH (Benjamini-Hochberg) method. GO, Gene Ontology. See also Figure S2 and Tables S4, S5, S6, and S7.

To explore the potential for cooperativity of USF2 and PLAG1 in binding additional regulatory elements across the genome we performed cross-linking immunoprecipitation followed by massively parallel next-generation sequencing (ChIP-seq) on two different clones (#4 and #5) of K562 cells co-overexpressing USF2 and FLAG-PLAG1-S (Figure S2). The chromatin was sheared to <500 bp fragments, the efficiency of pull-down was validated by ChIP followed by a western blot experiment and subsequent ChIP-seq fingerprint plot analysis (Figures S2A–S2D) (Ramirez et al., 2016). ChIP-seq peak calling revealed that USF2 and PLAG1 occupied a total of 2,409 and 21,469 sites, respectively (Figure S2E). To evaluate the likely biological impact, we assayed for co-binding within a stringent 100 bp window and found a statistically significant association of USF2 binding with PLAG1 sites as the majority (56%) of USF2 regions (1,386 sites) were accompanied by PLAG1 binding (Figure 6A, p value 2.2 × 10^−16^ by Fisher's exact test; Table S1A). We noted that USF2-PLAG1 (PLAG1 + USF2) co-occupancy typically occurred at promoter regions, with ∼70% of peaks detected within 3 kb of a transcription start site (TSS) (Figure 6B). These peaks also show a strong distribution centered at the TSS supporting the role of USF2 and PLAG1 as TFs at these locations (Figure 6C). We next performed *de novo* motif discovery on sites bound by USF2 or PLAG1. Analysis of the USF2 bound sites uncovered the expected E-box motif for USF2 as the most significantly enriched, as well as a second, less frequent G-rich motif. The motif discovery for PLAG1 revealed not only the G-rich core consensus binding motif recognized by PLAG family members (Voz et al., 1998) but also identified an E-box containing consensus sequence bound with an equivalent frequency to the PLAG1 G-rich core consensus (Figure 6D). The motif distribution profile of the USF2 consensus at the center of PLAG1 peaks (Figure 6E) provide further support for the concept that PLAG1 can associate with regions of chromatin to which USF2 would be expected to show an affinity and echoes the preferential co-localization of these two factors at selected genomic loci as quantified above.

We next sought to explore whether USF2 and PLAG1 regulate HSC-specific genes and/or pathways. Using gene set enrichment analysis (GSEA) we created ranked lists with publicly available gene expression profiles assembled from sub-fractions of the human hematopoietic hierarchy ranging from primitive (Lin-CD34+) to mature (Lin+) (Beck et al., 2013) and then used the 621 genes associated with the most high-confidence USF2-PLAG1 co-bound promoters (peak scores >200) as the gene set. Interestingly, we found no significant enrichment of HSC-specific gene promoters being bound by the two factors (Figure S2F). Furthermore, the USF2-PLAG1 co-bound sites did not show a significant enrichment of a separate HSC stem/progenitor gene set (Ivanova et al., 2002) (Figure S2F). Despite this, however, we did observe promoter co-occupancy for a number of recognized HSC regulators, including *DNMT3A*, *SOCS2*, *HOXA1*, and *MSI2* (Figure 6F, Table S1A). Strikingly, MSI2 is the only factor in this list of promoters co-bound by USF2 and PLAG1-S that is documented as capable of expanding human HSC, and the identification of *MSI2* in this independent ChIP-seq experiment provides key support of our earlier findings in this study. Interestingly, upon Gene Ontology (GO) and Kyoto Encyclopedia of Genes and Genomes (KEGG) pathway analysis of those genes that show co-occupancy, there was an abundance of genes involved in autophagy, a pathway important for stem cell control and homeostasis in the blood system (Ho et al., 2017, Mortensen et al., 2011) (Figures 6G and 6H, Tables S1B and S1C). Finally, performing unbiased GSEA we also find that USF2-PLAG1 co-occupied genes are positively enriched for those involved in cell cycle, membrane transport, RNA processing, and infectious diseases (Table S1D). Together these results suggest that a subset of PLAG1-USF2 bound sites are in fact localized to promoter regions with relevance to stem cell regulation as well as other genes whose involvement in the function of these two factors will now be of interest to explore further. Thus, we show that in the HSC context the co-ordinate regulation by USF2 and PLAG1 of these genes, in conjunction with MSI2, provides complementary mechanisms that contribute to the maintenance of stemness features.

## Discussion

To date, we have little knowledge of the endogenous regulators of *MSI2* transcription in the human HSPC compartment. Given the powerful role MSI2 plays in promoting HSPC self-renewal, identifying *MSI2* activators and their mechanism of action is required before their regenerative potential can be fully harnessed. Here, we have identified USF2 and PLAG1 as being both necessary, and, together, sufficient for significant transcriptional activation of *MSI2*. This discovery advances the understanding of how stem cell fate is propagated via the master regulator MSI2. In addition to describing a role for USF2 and PLAG1, our study provides key insights into the nature of their regulatory action. We observed that PLAG1 and USF2 appear to function additively on the *MSI2* promoter, but synergistically on actual *MSI2* transcription, which implies additional regulatory mechanisms act on the state of native chromatin through which these factors associate to further elevate transactivation. Indeed, our findings indicate that the competency of PLAG1 binding is dominant over that of USF2 at the *MSI2* promoter. Of note, our ChIP-seq analysis identified the G-rich consensus sequence previously described for PLAG1 (Voz et al., 1998) but also reveals that PLAG1 occupancy is frequently found at sites containing or surrounding an E-box motif that is highly similar to that bound by USF2. These data offer the possibility that PLAG1 could be an important nucleator for subsequent USF2 binding, the precise mechanism of which will now require more in-depth exploration. To this point, the specific sequence, occupancy status, and, by extension, shape of genomic regions proximal to E-boxes are known to modulate USF binding specificity (Gordan et al., 2013). Thus, the possibility that PLAG1 binding at or proximal to E-box motifs establishes a local structural configuration that is favorable to USF2 binding presents an attractive subject for subsequent exploration. The feasibility of PLAG1 acting to facilitate a permissive local environment for USF2 binding is supported by recent work on the related PLAG family member PLAGL1, which has highlighted the potential for PLAGL1 to display aspects of “pioneer factor” behavior (Varrault et al., 2017).

Our attempts to measure a direct interaction between PLAG1 and USF2 by coIP did not detect such a relationship, but it is important to point out that a negative coIP result does not preclude PLAG1 and USF2 having direct physical interaction or indirect cooperativity. Indeed, transcriptional control mechanisms are often complex, involving myriad co-factor interactions, and occur in the context of highly structured chromatin and often with dynamic kinetics (Morgunova and Taipale, 2017). As such, dissection of the physical and mechanistic relationship between PLAG1 and USF2 at regions of co-regulation is beyond the scope of our study but presents a priority topic for future study. However, our identification of PLAG1 and USF2 as key mediators of *MSI2* regulation improves our understanding of the upstream mechanism that controls HSPC maintenance and expansion.

Our work also supplements the current understanding on the nature of USF2 action in HSPCs. USF2 is known to regulate a number of diverse biological processes but has also previously been shown to regulate HOXB4 expression in human hematopoietic cells, via association with the primitive cell enriched-TF NF-Ya (Giannola et al., 2000, Zhu et al., 2003). We found that USF2, when co-expressed with PLAG1, is bound to the promoter of *HOXA1*, which has been shown to promote self-renewal of murine HSPCs (Bach et al., 2010, Fabb et al., 2004). Thus, in addition to hinting that USF2 may have a larger potential to function in combination with other, more HSC-restricted factors in the regulation of stemness genes, these findings also suggest that USF2 may play a larger role in HOX gene regulation.

PLAG1 was initially discovered in human pleomorphic adenomas of the salivary gland as a proto-oncogene (Debiec-Rychter et al., 2001, Kas et al., 1997, Voz et al., 1998). Here, we have expanded the repertoire of PLAG1 functionality into the maintenance of HSPCs. We have shown that it is the selective expression of PLAG1 within the most primitive cells of the blood system (Gazit et al., 2013) that enables it to function in co-ordination with the ubiquitously expressed USF2, ensuring appropriate elevated expression of *MSI2* in these cells. Our findings position PLAG1 as a key transcriptional regulator of HSPC in its own right. We have shown that USF2 and PLAG1 work cooperatively, but the large pool of binding sites for PLAG1 that do not overlap with USF2 may regulate genomic targets beyond which we can study here. In combination with the markedly enriched expression of PLAG1 in HSCs, this may hint at a larger USF2-independent role for PLAG1 in the control of HSC function, a topic that will now be important to explore in future studies.

## Experimental Procedures

### *MSI2* Promoter Cloning and Screening for Associated TFs

A 3,238 bp 5′-flanking sequence upstream of the translation start site of human *MSI2* (transcript variants 1 and 4) was PCR amplified from BAC clone RP11-784M23 (Roswell Park Cancer Institute) and cloned upstream of firefly luciferase in pGL3-basic (Promega). The transcriptional start site is predicted to be 173 nucleotides upstream of the translational start site. 5′-Serial truncations were generated using primers listed in Table S2A. TFs predicted by MatInspector (Quandt et al., 1995) to bind the human 385 bp minimal *MSI2* promoter were retained for comparison against publicly available databases of global gene expression profiles of diverse hematopoietic subpopulations to facilitate a final selection of genes that are expressed in primitive mouse and human HSCs (Bagger et al., 2016, Heng and Painter, 2008, Jojic et al., 2013, Novershtern et al., 2011). For subsequent promoter mutagenesis screening we prioritized TFs that (1) had commercially available antibodies, (2) are expressed in K562s, (3) are known to be expressed in other stem cell types and/or to regulate their homeostasis, and (4) exhibit binding sites that are conserved in the *MSI2* promoter region shared between human and mouse. Predicted TF binding sites were mutated by rolling circle site-directed mutagenesis (Zheng et al., 2004). Primers and nucleotide substitutions are provided in Table S2B. Heatmap depicting putative *MSI2* regulating TFs and their expression in the mouse hematopoietic hierarchy was generated using IMMGEN's (Immunological Genome Project) online My GeneSet tool.

### Promoter Clone Transfections and Reporter Assays

Five-hundred nanograms of each clone were transiently co-transfected with 25 ng of pRL-TK (internal transfection control expressing *Renilla* luciferase) in K562 cells in triplicate. Luminescence was measured 24–36 hr after transfection using Dual-Luciferase-Assay Kit (Promega) in a FLUOstar Omega luminometer (BMG Labtech). Firefly luciferase activity was normalized to *Renilla* luciferase activity to adjust for transfection efficiency.

### Western Blots

Total protein was extracted from K562 cells using lysis buffer (50 mM Tris-HCl [pH 8], 150 mM NaCl, 1% NP-40, 0.5% sodium deoxycholate, 0.1% SDS, EDTA 2 mM) supplemented with protease inhibitor cocktail (Roche diagnostics). Lysate was spun at 14,000 rpm for 20 min on a refrigerated tabletop centrifuge. Total protein concentration was determined by Bradford assay using BSA as standard (Bio-Rad). Ten micrograms of total protein was resolved on 10% Bis-Tris PAGE. Resolved protein was transferred onto an immobilon-P polyvinylidene fluoride (PVDF) membrane (EMD Millipore) on transfer buffer (25 mM Tris, 250 mM glycine, and 15% methanol) using Trans-blot Turbo apparatus (Bio-Rad). Membrane was blocked for 1 hr using LI-COR blocking buffer (LI-COR Biosciences). Primary antibodies against MSI2 (#ab7614, Abcam) β-actin (catalog no. A5441 clone AC15, Sigma-Aldrich), were diluted in LI-COR blocking buffer (1:1000) and incubated for 1 hr at room temperature, washed three times at 15 min intervals by using 1× TBST buffer (50 mM Tris, 150 mM NaCl, 0.05% Tween 20 [pH 7.6]). IRDye 680 Goat Anti-Rabbit (catalog no. 926–32221, LI-COR Biosciences) and IRDye 800CW Goat anti-Mouse (catalog no. 926–32210, LI-COR Biosciences) secondary antibodies were diluted (1:15,000) in LI-COR blocking buffer and incubated for 1 hr at room temperature. The membrane was washed three times at 15 min interval using 1× TBST buffer. Blots were imaged using LI-COR Odyssey imaging station (LI-COR Biosciences).

### qRT-PCR

Total RNA was isolated using Trizol-LS reagent (Life Technologies). cDNA synthesis and qPCR were done using the same protocol as Rentas et al. (2016) using Roche UPL primer and probe sets specific to each of *PLAG1*, *β-ACTIN*, *MSI2*, and *CYP1B1* (Table S2C). *PLAG1* isoform-specific qPCR assays were purchased from IDT (PrimeTime Mini qPCR Assay, PLAG1-A assay ID, Hs.PT.58.26984634; PLAG1-B assay ID, Hs.PT.58.22864822). Fold change in transcript level was calculated according to the 2^−ΔΔCt^ method.

### Generation of PLAG1 and USF2 Independent Overexpression

Open reading frame cDNA clones for *PLAG1* (Clone Id: 30915361) and *USF2* (Clone Id: 5483523) were obtained from Mammalian Gene Collections (GE Healthcare). cDNAs were PCR amplified using PrimeSTAR Max enzyme and subcloned into EcoRI/XhoI (NEB) digested MSCV-PGK/GFP vector. Primers used were: USF2-sense, CAT GCA TGG AAT TCG CCA CCA TGG ACA TGC TGG ACC, USF2-antisense, CAT GCA TGC TCG AGT CAC TGC CGG GTG CC (EcoRI and XhoI sites are underlined). To generate 3xFLAG epitope-tagged versions of the predominantly expressed variants of PLAG1-B and PLAG1-S, the respective PCR products were generated and cloned into the shuttle vector p3xFLAG-CMV10 (Sigma-Aldrich) at EcoRI/KpnI (NEB) sites. FLAG-tagged clones were PCR amplified and subcloned into MSCV-PGK/GFP vector at EcoRI/XhoI sites using MfeI/SalI (NEB) compatible end ligation. Primers used were: FLAG-PLAG1-sense, GAT CGA TCC AAT TGA TGG ACT ACA AAG ACC, FLAG-PLAG1-antisense, CAT GCA TGG TCG ACC TAC TGA AAA GCT TGA (MfeI and SalI sites are underlined).

### Generation of PLAG1 and USF2 Overexpression and Knockdown Constructs

*PLAG1* isoforms and *USF2* were cloned into the bidirectional promoter pSMALB vector where the transgene is driven in one direction and mtagBFP (blue fluorescent protein [BFP], as the transduction marker) in the other (van Galen et al., 2014). For co-overexpression, 3xFLAG-*PLAG1-100* and *USF2* were cloned consecutively into pSMALB and separated by a P2A site so that BFP positivity marks cells co-overexpressing both transgenes. shRNAs against *PLAG1* and *USF2* were designed using the RNAi Central tool and ligated downstream of the H1 promoter in the modified cppt-PGK-EGFP-IRES-PAC-WPRE lentiviral expression vector (Hope et al., 2010). Lentivirus production and transduction of K562 or primary Lin- CB cells was done as previously reported (Rentas et al., 2016). Stable lines were propagated with biological replicate lines created for every condition from three or more distinct transductions.

For details of ChIP and all other procedures, see Supplemental Experimental Procedures.

### Isolation of Primitive Human Hematopoietic Cells and Flow Cytometry

All patient samples were obtained with informed consent and with the approval of the local human subject research ethics board at McMaster University.

## Author Contributions

S.B. and A.K.C. contributed equally. M.S.B. designed and performed experiments, interpreted results, and wrote the manuscript. S.B. assisted with ChIP-seq experiments, performed ChIP-seq analysis, and interpreted results. A.K.C. designed and performed experiments, interpreted results, and assisted with manuscript preparation. S.R. assisted with cord blood experiments and manuscript preparation. J.S.D. designed experiments and wrote the manuscript. K.J.H. supervised the project, designed experiments, interpreted results, and wrote the manuscript.

## Accession Numbers

The accession number for the ChIP-seq data reported in this paper is GEO: GSE111469.

## Supplemental Information

Document S1. Supplemental Experimental Procedures, Figures S1 and S2, and Tables S1–S3 and S5–S7

Table S4. List of Promoter Regions Co-bound within a 100 bp Window by PLAG1-S and USF2, Related to Figure 6

Document S2. Article plus Supplemental Information
