# Supplementary material for: PLAG1 and USF2 Co-regulate Expression of Musashi-2 in Human Hematopoietic Stem and Progenitor Cells
Source: Stem Cell Reports. 2018 Apr 10;10(4):1384–97. doi: 10.1016/j.stemcr.2018.03.006 (PMC5998603; doi:10.1016/j.stemcr.2018.03.006)
Supplement: Document S1. Supplemental Experimental Procedures, Figures S1 and S2, and Tables S1–S3 and S5–S7 [file mmc1.pdf]

Stem Cell Reports, Volume *10*

Supplemental Information

PLAG1 and USF2 Co-regulate Expression of Musashi-2 in Human Hematopoietic Stem and Progenitor Cells

Muluken S. Belew, Sonam Bhatia, Ava Keyvani Chahi, Stefan Rentas, Jonathan S. Draper, and Kristin J. Hope

Figure S1

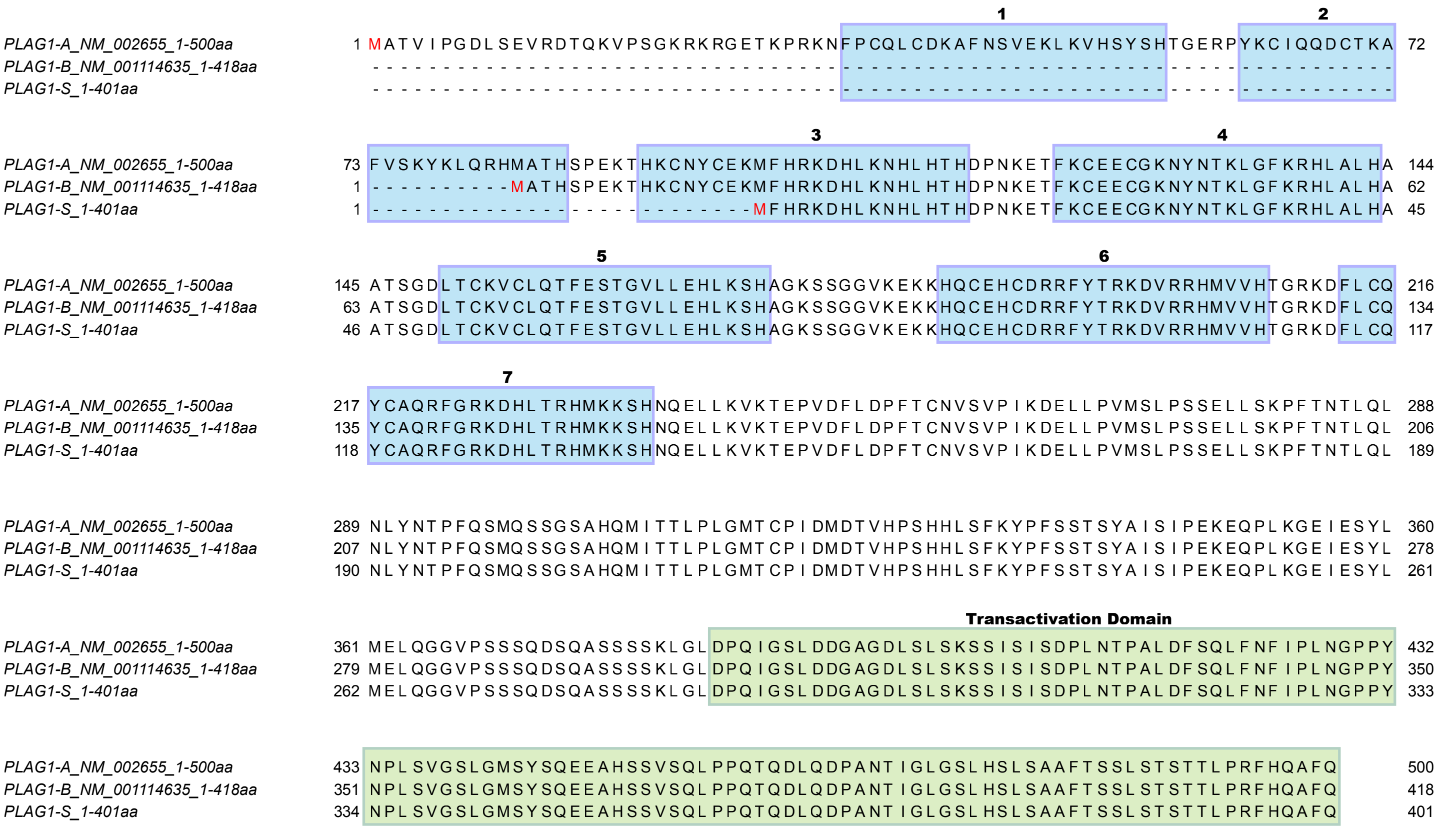

Figure S1. Sequence alignment of human PLAG1 isoforms. Related to Figure 2.
Shown is the alignment of the three PLAG1 isoforms and the location and number of Zn finger domains (blue boxes), transactivation domain (green box) and alternative start sites for PLAG1 isoforms (red).

Figure S2

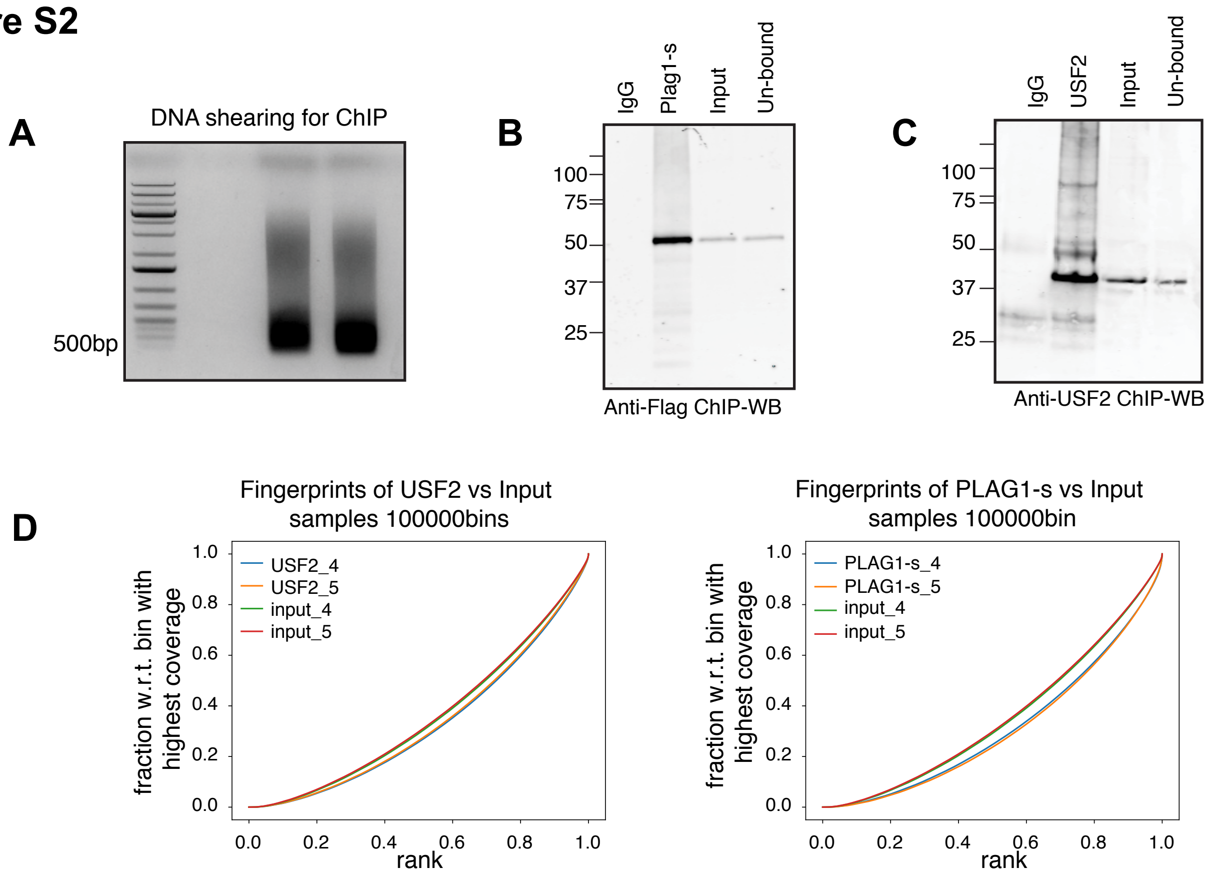

E Summary of peak calling

	FLAG_4	FLAG_5	USF2_4	USF2_5	Combined_input
Total tags	47474420	45001917	50765653	48829621	89027706
Total tags after filtering	47474413	45001910	50765644	48829599	88476945
number of peaks	37574	29889	9338	4369	
**combined common peaks**	**21469**		**2409**		

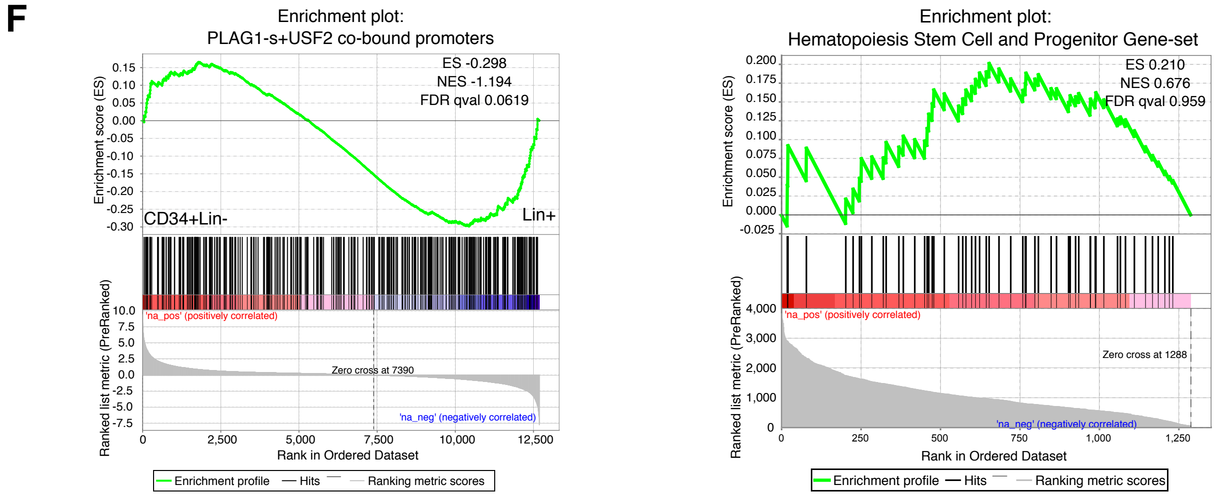

Figure S2. ChIP-seq experiments for PLAG1-s and USF2. Related to Figure 6.
(A.) Shearing profiles of the chromatin that was immunoprecipitated for ChIP-seq experiments. (B. and C.) ChIP-western blot (ChIP-WB) profiles to assess the efficiency of chromatin pull down prior to sequencing by (B.) anti-Flag antibody for PLAG1-s ChIP and (C.) anti-USF2 antibody for USF2 ChIP. (D.) Fingerprint plots showing the sequencing read coverage distribution across 100,000 randomly selected genomic bins for inputs as well as USF2, and PLAG1-s ChIP samples suggests that PLAG1-s ChIP was more efficient compared to USF2 ChIP (E.) Summary of peak calling for both individual replicates from K562 co-overexpression clones #4 and #5 for PLAG1-s and USF2 ChIP experiments. Peaks that were common to the two replicates (combined common peaks) were used for all downstream analysis. (F.) GSEA for PLAG1-s+USF2 co-occupied promoters against a ranked list of genes based on expression from Lin- to Lin+ populations (left) GSEA for PLAG1-s+USF2 co-occupied sites using the hematopoietic stem/progenitor gene set (right).

Table S1: Promoter fragment truncation and deletion primer sequences. Related to Figure 1. Same reverse primer was used in combination with the respective forward primer sequence to generate full length and truncation clones. Underlined are the XhoI and HindIII restriction sites in the forward and reverse sequences, respectively.

Promoter clone	Forward primer sequence	Reverse primer sequence
-3238-Luc	CTAGCTAGCTCGAGATGGAGAGCAGAAAGGCACG	CTAGCTAGAAGCTTATCTGAGCCCCCCCGCCCCC
-2251-Luc	CTAGCTAGCTCGAGGTGAGTTTGGCACCTCTGAG	
-1764-Luc	CTAGCTAGCTCGAGGTGAGTTTGGCACCTCTGAG	
-1188-Luc	CTAGCTAGCTCGAGAGGGGTTTCCTGAAATAACC	
-1013-Luc	CTAGCTAGCTCGAGCTTTCTCGCCACTGGAATCG	
-788-Luc	CTAGCTAGCTCGAGCGGGGGCCGCCAAAGGAG	
-588-Luc	CTAGCTAGCTCGAGGAGGAGCCGCAGCAAGC	
-203-Luc	CTAGCTAGCTCGAGTGAGCTAAGCCGAGCCC	
-138-Luc	CTAGCTAGCTCGAGATTCGGAGGAGCCCGG	
d588-203-Luc	TGAGCTAAGCCGAGCCC	ACCCGGACCTGGGAGAAG

Table S2: Transcription factor binding site mutagenesis primers. Related to Figure 1.

Transcription Factor	Predicted Number of Binding Sites	Consensus Binding Sequence	Substitution	Forward Primer	Reverse Primer
Upstream stimulatory factor-2 (USF2)	3	CANNTG	GTNNGA	CCCGCCCCCCGCAGCCTCTGGAATCCAGGGAAGTCGGG<br>TCTAGAACTCCGCCCTCCGGACGCCGGGGAGGAGGG<br>GAGCTAAGCCGAGCCTCCGGATGACGGCTCTCGCCG	CCCGACTTCCCTGGATTCCAGAGGCTGCGGGGGGCGG<br>CCCTCCTCCCCGGCGTCCGGAGGGCGGAGTTCTAGA<br>CGGCGAGAGCCGTCATCCGGAGGCTCGGCTTAGCTC
Nuclear factor Y-A (NFYA)	1	GGCATTGGTT	TCAGCTGTAT	GGTGCGTGACGTCACCTCAGCTGTATACACGACGTTCTAGAAC	GTTCTAGAACGTCGTGTATACAGCTGAGGTGACGTCACGCACC
cAMP responsive element binding protein (CREB)	1	TGACGT	TTAAGT	CGCACGGGGGTGCGTTAATTTACCGGCATTGGTTACA	TGTAACCAATGCCGGTAAATTAACGCACCCCCGTGCG
Glucocorticoid Nuclear Receptor (NR3C1/GRE)	1	GTTC	GCCC	CATTGGTTACACGACGCCCTAGAACTCCGCCCCA	TGGGGCGGAGTTCTAGGGCGTCGTGTAACCAATG
Microphthalmia-Associated Transcription Factor (MITF)	1	CATGTG	GATATA	CCCGCCCCCCGCAGCCGATATAATCCAGGGAAGTCGGG	CCCGACTTCCCTGGATTATATCGGCTGCGGGGGGCGGG
CCCTC-binding factor (CTCF)	5	GGGG	ATATT	CCCCCGATCCACCCGAATATCGCAGCCCATGTGAT<br>GTGGGTCCGGCCGTGATATTCCGAGTGTGCGCGCG<br>GTGCGCGCGCGGGCAGGCATATTCCGCACATATTTGCGTGACGTCACCGGC<br>GCCGGGGGGGCGGGGAATATTGGGTGTGCGAGGCAGC	ATCACATGGGCTGCGATATTCGGGTGGATCGGGGG<br>CGCGCGCACACTCGGAATATCACGGCCGGACCCAC<br>GCCGGTGACGTCACGCAAATATGTGCGGAATATGCCTGCCCGCGCGCGCAC<br>GCTGCCTCGCACACCCAATATTCCCCGCCCCCCCGGC
PR Domain Containing 5 (PRDM5)	2	AGGG	TCTT	CGTGCGCCGGGGAGGTCTTGGAGGAGGAGGAGGA<br>GCGGGGGGGGAGGAGGTCTTGGAGGAGGGAGCGGA	TCCTCCTCCTCCTCCAAGACCTCCCCGGCGCACG<br>TCCGCTCCCTCCTCCAAGACCTCCTCCCCCCCCGC
Zinc fingerDNA binding protein 148/89 (ZFP148/ZBP89)	10	C-rich: CCCC<br>G-rich: GGGG	C-rich: CGGT<br>G-rich: GCCA	CGCCTCCCCTCGGTCGATCCACCCGCGGTCCGCAGCCCAT<br>GTCGGGGTGCGCTCGGTCTCGCCCTGCGCCC<br>CCGGGGAGGAGGCCAAGGAGGAGGAGG<br>GGAGGAGATGGCCATGGGGAGGAGGAGGCCAAGAGGTGGGGAT<br>GGGATGGGCCGGGGCCACGGGGACGGGG<br>CGGGGACGGGGCCATGTGCGAGGCAGC<br>CCGGGCGGGGCCAAGGAGGAGGCCAAGGAGGGAGCG	ATGGGCTGCGGACCGCGGGTGGATCGACCGAGGGGAGGCG<br>GGGCGCAGGGCGAGACCGAGCGCACCCCGAC<br>CCTCCTCCTCCTTGGCCTCCTCCCCGG<br>ATCCCCACCTCTTGGCCTCCTCCTCCCCATGGCCATCTCCTCC<br>CCCCGTCCCCGTGGCCCCGGCCCATCCC<br>GCTGCCTCGCACATGGCCCCGTCCCCG<br>CGCTCCCTCCTTGGCCTCCTCCTTGGCCCCGCCCGG
Pleomorphic adenoma gene 1 (PLAG1)	3	GGGG	TGAT	GTGCGCCGGGGAGGATGATGAGTAATAGGAGGAGATGG<br>GGAGATGGGGGTATGGAGGAGGAATGAGAGAGGTGGGG<br>CAGAGAGATTCGTAATAGCCCGGGCGTGATGGAGGAGGAGGGGGAGGAGGGAGCG	CCATCTCCTCCTATTACTCATCATCCTCCCCGGCGCAC<br>CCCCACCTCTCTCATTCCTCCTCCATACCCCCATCTCC<br>CGCTCCCTCCTCATCATCCTCCTCCATCACGCCCGGGCTATTACGAATCTCTCTG
Myc associated zinc finger protein1 (MAZ1)	7	GGGAGGGNNNN<br>CCCTCCCNNNN	TGTATGTNTNT<br>ACATACANANA	AAGCTCCCCGCGACTCACATACACAGATCCACCCGCC<br>GGGGTGCGCTCCACATAGACATGCGCCCTGCC<br>GGAGGCGGGGTCACATACACCCGCGGGGTTC<br>CCACGTGCGCCGTGTATGATGGGGAGGAGGAG<br>GGGAGGAGGAGGTGTATATGTGGGGATGGGC<br>GGGAGGAGGAGGTGTATGATGGAGCGGAGATC<br>TTTTGGGGGTGGGGTGTCTGTGGGGCTCAGAT	GGCGGGTGGATCTGTGTATGTGAGTCGCGGGGAGCTT<br>GGCAGGGCGCATGTCTATGTGGAGCGCACCCC<br>GAACCCCGCGGGTGTATGTGACCCCGCCTCC<br>CTCCTCCTCCCCATCATACACGGCGCACGTGG<br>GCCCATCCCCACATATACACCTCCTCCTCCC<br>GATCTCCGCTCCATCATACACCTCCTCCTCCC<br>ATCTGAGCCCCACAGACACCCCACCCCCAAAA

Table S3. qPR-PCR Primers and Universal Probe Library Number for MSI2 and CYP1B1. Related to Figure 2-5.

Gene	Accession	Primer sequences	Length	Position	Tm	%GC	UPL#
MSI2 isoform 1	NM_138962.2	ggcagcaagaggatcagg<br>ccgtagagatcggcgaca	18<br>18	947 - 964<br>1016 - 1033	59<br>60	61<br>61	26
CYP1B1	NM_000104.3	acgtaccggccactatcact<br>ctcgagtctgcacatcagga	20<br>20	1359 - 1378<br>1450 - 1469	59<br>60	55<br>55	20

Table S5: KEGG Pathway Analysis of genes co-bound by PLAG1-S and USF2. Related to Figure 6.

ID	Description	GeneRatio	BgRatio	pvalue	p.adjusted	qvalue	geneID	Count
hsa04142	Lysosome	22/436	123/7317	2.78E-06	7.79E-04	6.85E-04	3916/9114/57192/4758/2548/285362/2799/9179/1509/1201/1508/51606/23062/1200/1497/4074/8722/2629/5538/8905/3425/427	22
hsa05120	Epithelial cell signaling in Helicobacter pylori infection	15/436	68/7317	7.94E-06	1.11E-03	9.78E-04	9114/528/5336/8992/9550/529/155066/998/51606/6300/5599/102/4792/6714/3725	15
hsa04933	AGE-RAGE signaling pathway in diabetic complications	18/436	99/7317	1.78E-05	1.66E-03	1.46E-03	581/5581/5336/998/6776/5296/1729/596/6300/5599/4893/5054/4846/4088/5594/595/7423/3725	18
hsa04917	Prolactin signaling pathway	13/436	70/7317	2.09E-04	1.46E-02	1.28E-02	8835/2592/6776/5296/6300/5599/25759/4893/5594/2309/595/6714/894	13
hsa04721	Synaptic vesicle cycle	12/436	63/7317	2.84E-04	1.59E-02	1.40E-02	9114/528/8992/8775/9550/529/155066/51606/2054/774/5864/594855	12
hsa04140	Autophagy - animal	18/436	128/7317	5.39E-04	2.15E-02	1.89E-02	3916/64121/64422/1509/1508/29982/11337/5296/596/5599/4893/10325/55102/5594/55062/22863/9474/54541	18
hsa05110	Vibrio cholerae infection	10/436	50/7317	6.06E-04	2.15E-02	1.89E-02	9114/528/5336/8992/9550/529/155066/51606/60/375	10
hsa04370	VEGF signaling pathway	11/436	59/7317	6.14E-04	2.15E-02	1.89E-02	5336/998/5296/6300/25759/4893/4846/8877/5594/7867/6714	11
hsa04966	Collecting duct acid secretion	7/436	27/7317	7.95E-04	2.47E-02	2.18E-02	9114/528/8992/9550/529/155066/10723	7
hsa04137	Mitophagy - animal	11/436	65/7317	1.42E-03	3.62E-02	3.18E-02	79735/11337/5599/4893/2309/665/9474/55288/6714/51024/3725	11
hsa05131	Shigellosis	11/436	65/7317	1.42E-03	3.62E-02	3.18E-02	998/1729/6300/5599/3688/5594/9474/4792/6714/60/7414	11
hsa04931	Insulin resistance	15/436	107/7317	1.59E-03	3.70E-02	3.26E-02	6945/5581/92579/133522/5296/5599/4846/5106/10891/5770/1374/6720/4792/79660/10999	15

Table S6: GO Pathway Analysis of genes co-bound by PLAG1-S and USF2. Related to Figure 6.

ID	Description	GeneRatio	BgRatio	pvalue	p.adjusted	qvalue	geneID	Count
GO:0006914	autophagy	57/983	428/16672	6.24E-09	2.96E-05	2.84E-05	LAMP1/ATP6V0D1/MCOLN1/ULK3/RRAGC/ATG3/MTDH/TRAPPC8/CTSD/ATP6V1C1/VPS26A/ATP6V0E1/ATP6V1G1/RAB3GAP2/TBC1D17/ATP6V1E1/ATP6V0E2/LAMTOR1/CLN3/WDR6/ATP6V1H/NRBF2/VPS37B/VPS11/U2AF2/PIK3R2/BCL2/FBXL2/MTM1/PIP4K2C/MAPK8/GAPDH/VPS33A/VPS16/RNF185/TICAM1/GBA/TPCN1/RRAGB/ATG2B/EXOC1/CDK5R1/LAMTOR4/PIM2/WIPI1/PPARGC1A/BNIP3L/ATG14/FUNDC2/ATG5/MAP1S/KIAA1324/SREBF1/SPTLC2/NEDD4/FIS1/UBC	57
GO:0016236	macroautophagy	35/983	234/16672	3.46E-07	8.20E-04	7.87E-04	ATP6V0D1/MCOLN1/RRAGC/ATG3/TRAPPC8/ATP6V1C1/VPS26A/ATP6V0E1/ATP6V1G1/RAB3GAP2/ATP6V1E1/ATP6V0E2/LAMTOR1/CLN3/ATP6V1H/MTM1/PIP4K2C/MAPK8/GAPDH/VPS33A/VPS16/GBA/RRAGB/ATG2B/EXOC1/CDK5R1/LAMTOR4/WIPI1/BNIP3L/ATG14/ATG5/KIAA1324/SPTLC2/NEDD4/UBC	35
GO:0010506	regulation of autophagy	35/983	261/16672	4.55E-06	7.19E-03	6.90E-03	ATP6V0D1/RRAGC/MTDH/ATP6V1C1/VPS26A/ATP6V0E1/ATP6V1G1/RAB3GAP2/ATP6V1E1/ATP6V0E2/CLN3/WDR6/ATP6V1H/NRBF2/U2AF2/PIK3R2/BCL2/FBXL2/MTM1/PIP4K2C/MAPK8/GAPDH/TICAM1/GBA/TPCN1/RRAGB/EXOC1/CDK5R1/PIM2/BNIP3L/ATG14/KIAA1324/SREBF1/SPTLC2/NEDD4	35
GO:0016241	regulation of macroautophagy	21/983	123/16672	9.67E-06	1.15E-02	1.10E-02	ATP6V0D1/ATP6V1C1/VPS26A/ATP6V0E1/ATP6V1G1/RAB3GAP2/ATP6V1E1/ATP6V0E2/CLN3/ATP6V1H/MTM1/PIP4K2C/MAPK8/GAPDH/GBA/EXOC1/CDK5R1/BNIP3L/KIAA1324/SPTLC2/NEDD4	21
GO:0048511	rhythmic process	38/983	310/16672	1.51E-05	1.43E-02	1.38E-02	BAX/NR1D2/DYRK1A/VGF/TEF/BHLHE41/HNRNPD/DBP/NR1D1/SRD5A1/BCL2/MAPK8/CRY1/RELB/ADNP/HLF/SERPINE1/NOS3/KLF10/CDK5R1/ANG/BMPR1B/CSF2/TIMELESS/DDX5/PER3/FOXO3/PPARGC1A/CPT1A/RORA/TOP1/SREBF1/CHRNA7/NR5A1/JUND/MSH4/SOD1/JUN	38
GO:0007033	vacuole organization	28/983	209/16672	4.06E-05	2.95E-02	2.83E-02	MCOLN1/PDCD6IP/GAA/ATG3/TRAPPC8/ARFGEF2/RAB3GAP2/LAMTOR1/CLN3/HPS1/VPS37B/VPS11/MTM1/PIP4K2C/TPP1/VPS33A/VPS16/HOOK2/PPT1/ATG2B/WIPI1/STX6/P2RX7/ATG14/ATG5/KIAA1324/CLN6/ARF1	28
GO:0051452	intracellular pH reduction	11/983	45/16672	4.50E-05	2.95E-02	2.83E-02	ATP6V0D1/ATP6V1C1/ATP6V0E1/ATP6V1G1/ATP6V1E1/ATP6V0E2/CLN3/ATP6V1H/BCL2/PPT1/CLN6	11
GO:0045851	pH reduction	11/983	46/16672	5.60E-05	2.95E-02	2.83E-02	ATP6V0D1/ATP6V1C1/ATP6V0E1/ATP6V1G1/ATP6V1E1/ATP6V0E2/CLN3/ATP6V1H/BCL2/PPT1/CLN6	11
GO:0048025	negative regulation of mRNA splicing, via spliceosome	7/983	19/16672	6.53E-05	2.95E-02	2.83E-02	DYRK1A/U2AF2/PTBP1/SFSWAP/SRSF6/SRSF7/HNRNPA2B1	7
GO:0007034	vacuolar transport	33/983	272/16672	6.62E-05	2.95E-02	2.83E-02	AP5Z1/LAMP1/VPS50/AP4M1/MICALL2/TRAPPC8/VPS26A/RAB29/TBC1D17/SPAG9/SNX8/CLN3/SNX2/RAB21/VPS37B/VPS11/MTM1/GPRASP1/VPS33A/M6PR/VPS16/SNX16/HOOK2/MAPK1/SNX9/WIPI1/STX6/ATG14/SRC/RHOB/NEDD4/ARF1/UBC	33
GO:0042594	response to starvation	22/983	150/16672	6.84E-05	2.95E-02	2.83E-02	RRAGC/FNIP1/SLC38A2/HMGCL/UCP2/SRD5A1/BCL2/CAD/COMT/BCAS3/GBA/RRAGB/KLF10/PPARGC1A/SEH1L/ATG14/ATG5/	22

							KIAA1324/NUAK2/SREBF1/AACS/JUN	
GO:0071417	cellular response to organonitrogen compound	48/983	462/16672	9.61E-05	3.71E-02	3.56E-02	ATP6V0D1/SOCS2/RRAGC/MGARP/PRKAR2B/ATP6V1C1/ATP6V0E1/ATP6V1G1/APEX1/ATP6V1E1/ATP6V0E2/LAMTOR1/OSBPL8/HNRNPD/ATP6V1H/PLA2G1B/STAT5A/UCP2/SRD5A1/PIK3R2/RAB31/COL6A1/DIAPH1/DNMT3A/GCLC/AGTRAP/PDK2/KLF15/RRAGB/KLF10/MAPK1/LAMTOR4/KLF4/FER/HDAC5/FOXO3/FGF21/PPARGC1A/BAIAP2L1/CAPN10/PTPN1/COL16A1/SLC34A1/PRKAR1A/SREBF1/PDE2A/SRC/SOD1	48
GO:1901652	response to peptide	45/983	425/16672	1.02E-04	3.71E-02	3.56E-02	ATP6V0D1/SOCS2/MGARP/PRKAR2B/ATP6V1C1/VGF/BSG/ATP6V0E1/ATP6V1G1/APEX1/ATP6V1E1/ATP6V0E2/OSBPL8/ATP6V1H/PLA2G1B/STAT5A/UCP2/SRD5A1/PIK3R2/RAB31/GCLC/CAD/AGTRAP/KHK/CRY1/PDK2/KLF15/KLF10/MAPK1/KLF4/FER/CACYBP/HDAC5/FOXO3/FGF21/BAIAP2L1/CAPN10/PTPN1/SLC34A1/PRKAR1A/SREBF1/NFKBIA/SRC/SRSF6/JUND	45
GO:1901653	cellular response to peptide	35/983	304/16672	1.17E-04	3.97E-02	3.81E-02	ATP6V0D1/SOCS2/MGARP/PRKAR2B/ATP6V1C1/ATP6V0E1/ATP6V1G1/APEX1/ATP6V1E1/ATP6V0E2/OSBPL8/ATP6V1H/PLA2G1B/STAT5A/UCP2/SRD5A1/PIK3R2/RAB31/GCLC/AGTRAP/PDK2/KLF15/KLF10/MAPK1/KLF4/FER/HDAC5/FOXO3/FGF21/BAIAP2L1/CAPN10/PTPN1/PRKAR1A/SREBF1/SRC	35
GO:0008333	endosome to lysosome transport	10/983	42/16672	1.26E-04	4.00E-02	3.84E-02	VPS11/MTM1/GPRASP1/VPS33A/M6PR/VPS16/SNX16/HOOK2/ATG14/RHOB	10
GO:1903312	negative regulation of mRNA metabolic process	9/983	35/16672	1.46E-04	4.32E-02	4.14E-02	DYRK1A/U2AF2/PTBP1/CCNT1/SFSWAP/SRSF6/SRSF7/TOB1/HNRNPA2B1	9
GO:0000082	G1/S transition of mitotic cell cycle	28/983	227/16672	1.74E-04	4.53E-02	4.35E-02	BAX/CACUL1/HINFP/CDK7/APEX1/EIF4E/BCL2/NACC2/GSPT1/PRMT1/RPA1/ITGB1/MEPCE/JADE1/KANK2/PIM2/PLK5/CNOT4/CCNE1/CCND1/CUL4B/CDC25C/MCM5/MCM2/CTDSP2/CCND2/PLAGL1/UBC	28
GO:0007040	lysosome organization	11/983	52/16672	1.83E-04	4.53E-02	4.35E-02	GAA/LAMTOR1/CLN3/HPS1/VPS11/TPP1/HOOK2/PPT1/P2RX7/CLN6/ARF1	11
GO:0080171	lytic vacuole organization	11/983	52/16672	1.83E-04	4.53E-02	4.35E-02	GAA/LAMTOR1/CLN3/HPS1/VPS11/TPP1/HOOK2/PPT1/P2RX7/CLN6/ARF1	11
GO:0007035	vacuolar acidification	6/983	16/16672	1.99E-04	4.53E-02	4.35E-02	ATP6V0E1/ATP6V0E2/CLN3/ATP6V1H/PPT1/CLN6	6
GO:0050686	negative regulation of mRNA processing	8/983	29/16672	2.00E-04	4.53E-02	4.35E-02	ATP6V0E1/ATP6V0E2/CLN3/ATP6V1H/PPT1/CLN6	8

Table S7: Reactome GSEA of genes co-bound by PLAG1-S and USF2. Related to Figure 6.

NAME	SIZE	ES	NES	NOM p-val	FDR q-val	FWER p-val	RANK AT MAX	LEADING EDGE
REACTOME_CELL_CYCLE	25	0.69933075	1.444864	0	0.092173874	0.088	257	tags=36%, list=20%, signal=44%
REACTOME_TRANSMEMBRANE_ TRANSPORT_OF_SMALL_MOLECULES	37	0.6706475	1.4014298	0.002	0.11639127	0.196	176	tags=32%, list=14%, signal=36%
REACTOME_CELL_CYCLE_MITOTIC	20	0.6407614	1.2703049	0.07214429	0.503188	0.78	257	tags=35%, list=20%, signal=43%
REACTOME_MRNA_PROCESSING	22	0.6112415	1.2238253	0.118236475	0.6260868	0.924	319	tags=41%, list=25%, signal=53%
REACTOME_HIV_INFECTION	19	0.605965	1.2200736	0.125	0.51733893	0.934	309	tags=47%, list=24%, signal=61%
REACTOME_METABOLISM_ OF_PROTEINS	37	0.5639318	1.1745437	0.12825651	0.6692175	0.99	343	tags=41%, list=27%, signal=54%
REACTOME_METABOLISM_ OF_CARBOHYDRATES	17	0.56408656	1.122007	0.27108434	0.8701489	1	180	tags=29%, list=14%, signal=34%
REACTOME_SLC_MEDIATED_ TRANSMEMBRANE_TRANSPORT	24	0.5438985	1.106202	0.284	0.8493914	1	144	tags=21%, list=11%, signal=23%
REACTOME_PHOSPHOLIPID_ METABOLISM	18	0.5415519	1.0580957	0.4084507	1	1	390	tags=44%, list=30%, signal=63%
REACTOME_GENERIC_ TRANSCRIPTION_PATHWAY	16	0.5298262	1.0539083	0.4248497	0.95460856	1	305	tags=31%, list=24%, signal=40%
REACTOME_METABOLISM_ OF_LIPIDS_AND_LIPOPROTEINS	39	0.49715915	1.0450431	0.388	0.91145444	1	390	tags=41%, list=30%, signal=57%
REACTOME_TRANSCRIPTION	18	0.52930707	1.0423437	0.40480962	0.8432317	1	394	tags=33%, list=31%, signal=47%

REACTOME_PLATELET_ ACTIVATION_SIGNALING_ AND_AGGREGATION	15	0.5285818	1.0418788	0.42566192	0.77960515	1	330	tags=27%, list=26%, signal=35%
REACTOME_ADAPTIVE_ IMMUNE_SYSTEM	26	0.513643	1.0310123	0.452	0.76632285	1	344	tags=38%, list=27%, signal=51%
REACTOME_PROCESSING_ OF_CAPPED_INTRON_ CONTAINING_PRE_MRNA	19	0.5146221	1.0180796	0.49	0.75990134	1	319	tags=32%, list=25%, signal=41%
REACTOME_HEMOSTASIS	31	0.43617868	0.9102693	0.726	1	1	353	tags=26%, list=27%, signal=35%
REACTOME_SIGNALING_BY_GPCR	23	0.4300659	0.8785485	0.73547095	1	1	330	tags=26%, list=26%, signal=34%
REACTOME_IMMUNE_SYSTEM	53	0.399912	0.85586494	0.888	1	1	570	tags=47%, list=44%, signal=81%
REACTOME_SIGNALLING_BY_NGF	20	0.42523456	0.85515	0.7755511	1	1	570	tags=55%, list=44%, signal=97%
REACTOME_MRNA_SPLICING	16	0.4288823	0.84179723	0.77911645	0.9739479	1	319	tags=25%, list=25%, signal=33%
REACTOME_GPCR_ DOWNSTREAM_SIGNALING	18	0.41564652	0.81756485	0.8266129	0.9627115	1	330	tags=28%, list=26%, signal=37%
REACTOME_INNATE_IMMUNE_SYSTEM	21	0.30153662	0.60955846	0.9839357	1	1	747	tags=57%, list=58%, signal=134%
REACTOME_DEVELOPMENTAL_BIOLOGY	24	0.27081522	0.54898703	0.986	1	1	869	tags=71%, list=67%, signal=214%
REACTOME_NEURONAL_SYSTEM	15	0.26906207	0.526231	0.9919355	0.993899	1	712	tags=47%, list=55%, signal=103%

Supplemental Experimental Procedures

Co-Immunoprecipitation

1x10^7 Cells were lysed in RIPA buffer containing protease inhibitors and incubated with rotation overnight at 4°C with 2μg anti-USF2 (SantaCruz, #sc-293443) or anti-mouse IgG (SantaCruz) and Protein G dynabeads. The precipitated complex was washed with RIPA buffer and coimmunoprecipitated proteins were eluted in 2x SDS Loading buffer and analyzed by Western Blot. Similar experiments were performed with 1% PFA cross-linked cells and anti-Flag (Sigma, F1804).

Chromatin Immunoprecipitation (ChIP)

ChIP-qPCR was done as per Carey et al with slight modifications (Carey et al., 2009). Briefly, cells were cross-linked, lysed and nuclei subjected to M220 focused-ultra sonicator (Covaris) to generate sheared chromatin fragments of ~500 bp. 5μg anti-USF2 (SantaCruz, #sc-862), anti-PLAG1 (GenTex, # GTX124217) or anti-rabbit IgG (SantaCruz) were added per 1000μL of IP volume followed by precipitation of chromatin-antibody complex by Protein G dynabeads (Invitrogen). Immunoprecipitated DNA was extracted using phenol/chloroform, quantified and subjected to qPCR. Primers and probes were designed by the Universal Probe Library (UPL) probe finder tool (Roche) using input sequence comprising 588 bp upstream of the MSI2 TSS. For the promoter region we used UPL probe #63 and forward and reverse primers as follows: CGCTCGCAGAGAGATTCG; GAGATCTCCGCTCCCTCCT. ChIP fold enrichment was calculated using the $2^{-\Delta\Delta Ct}$ method (Schmittgen and Livak, 2008).

For ChIP-seq, 1x10^8 cells K562 cells co-overexpressing Flag-PLAG1-S and USF2 were cross-linked in 1% PFA and excess PFA was washed with glycine, cells were lysed in

RIPA buffer (50mM Tris-Cl pH7.45, 150mM NaCl, 0.1% SDS, 2% NP-40, 1% Sodium deoxycholate) containing protease inhibitors and nuclei were subjected to probe sonication. Two percent of each sample was used for Input sequencing. Chromatin was diluted in dilution buffer (50mM Tris-Cl pH7.45, 150mM NaCl) to get a final SDS concentration of 0.025% to assist in immunoprecipitation (IP). Twenty μg anti-USF2 (SantaCruz, #sc-293443) or anti-Flag (Sigma, F1804) and Protein G dynabeads were used for IP. The precipitated complex was washed in low (50mM Tris-Cl, pH 7.45, 250mM NaCl, 2mM EDTA, 0.1%SDS w/v, 1% Triton X-100 v/v) and high salt buffer (same as low salt buffer except use 500mM NaCl) and chromatin was eluted in TE buffer at 65°C before reverse cross-linking with Proteinase K (NEB, P8107S). Sequencing libraries were prepared using NEBNext® Ultra™ II DNA Library Prep Kit for Illumina® (NEB, E7645S) and sequencing as 50 bps single ended reads was performed on Illumina HiSeq 1500 at a depth of 55 million reads per input and 42.5 million reads per IP.

ChIP-seq analysis

Sequencing reads were quality controlled using fastQC, and the adaptors were removed using cutadapt software using the illumina universal primer sequence AGATCGGAAGAGCACACGTCTGAACTCCAGTCAC. The reads were aligned to human genome assembly GRCh38 using hisat2 aligner (version hisat2-2.0.4) (Kim et al., 2015) and duplicates were removed using samtools (Li et al., 2009). Fingerprint plots were generated using deepTools (Ramirez et al., 2016) to check efficiency of the ChIP experiments. Samtools merge was used to merge the input files to create a

master background files, and MACS2 (Feng et al., 2011) was used to called peaks from the individual ChIP replicates using the merged input files. The following peak calling parameters were used:

macs2 callpeak -t ChIP_file1.bam -c Input_merged.bam -f BAM -g hs –n ChIP_file1_peaks -B -s 50 --bw 150 -q 0.01 --outdir

Since the IP efficiency was lower for USF2 the peaks were called using the following less stringent parameters:

macs2 callpeak –t USF2_ChIP_file1.bam -c Input_merged.bam -f BAM -g hs -n ChIP_file1_peaks -B -s 50 --bw 150 -q 0.05 –m 2 50 --outdir

Bedtools intersect (Quinlan and Hall, 2010) was used to identify peaks common to the two replicates and were used for all downstream analysis. Peak files were annotated using HOMER (Heinz et al., 2010) annotatepeaks.pl script using hg38 as the background. HOMER's mergepeaks function was used to identify PLAG1-S and USF2 co-bound targets within 100bps from each other. findMotifsGenome.pl was used for motif analysis with the following parameters -size 200 –mask. Motif distribution profiles were generated using the HOMER function annotatePeaks.pl with the USF2 consensus motif file and the second G-rich region identified as being the PLAG1-S consensus. The R-packages ChIPSeeker (Yu et al., 2015), and clusterProfiler (Yu et al., 2012) were used to perform peak distribution and pathway analysis. Significance of any enriched pathways and GO processes was addressed by setting adjusted pvalueCutoff to 0.05, and by adjusting the p-values using Benjamini & Hochberg (BH) method that is built into the clusterProfiler package. Gene-set enrichment analyses were performed using the GSEA tool with 500-1500 permutations.

Cord blood CD34+ cell isolation, lentiviral transduction and *in vitro* culture

FACS isolated Lin^- $CD34^+$ cells were prestimulated for 8-12 hours in StemSpan medium (StemCell Technologies) supplemented with growth factors Interleukin 6 (IL-6; 20 ng/ml, Peprotech), Stem cell factor (SCF; 100 ng/ml, R&D Systems), Flt3 ligand (FLT3-L; 100 ng/ml, R&D Systems) and Thrombopoietin (TPO; 20 ng/ml, Peprotech). Cells were transduced at an MOI of 50-100 with pSMALB lentiviral particles encoding 3xFLAG-PLAG1-p2A-USF2 (PLAG1+USF2) or firefly luciferase (Vector). BFP+ cells were purified using a MoFlo XDP (Beckman Coulter) 72hrs post-transduction. For *in vitro* culture, cells were seeded at a density of 1×10^5 cells/ml in IMDM 10% FBS supplemented with IL-6 (10 ng/ml), SCF (50 ng/ml), FLT3-L (50 ng/ml), and TPO (20 ng/ml) and counted and analyzed by flow cytometry for CD34 positivity at day 3 and 7. Flow cytometry data was analyzed by Flowjo (Tree star). Immunocytochemistry staining, imaging and analysis for MSI2 and CYP1B1 protein levels was done as previously reported (Rentas et al., 2016).

Statistical analysis

All statistical analyses were performed using PRISM (Graphpad software, Inc). Where appropriate statistical significance was estimated using non-parametric two-tailed t-test for pairwise comparisons or 1-way ANOVA multiple comparisons of control and test transfections/transductions. Data shown as mean ±SEM. * $p<0.05$, ** $p<0.01$, *** $p<0.001$

Supplemental References

Carey, M.F., C.L. Peterson, and S.T. Smale. 2009. Chromatin immunoprecipitation (ChIP). *Cold Spring Harbor protocols* 2009:pdb prot5279.

Feng, J., T. Liu, and Y. Zhang. 2011. Using MACS to identify peaks from ChIP-Seq data. *Curr Protoc Bioinformatics* Chapter 2:Unit 2 14.

Heinz, S., C. Benner, N. Spann, E. Bertolino, Y.C. Lin, P. Laslo, J.X. Cheng, C. Murre, H. Singh, and C.K. Glass. 2010. Simple combinations of lineage-determining transcription factors prime cis-regulatory elements required for macrophage and B cell identities. *Mol Cell* 38:576-589.

Kim, D., B. Langmead, and S.L. Salzberg. 2015. HISAT: a fast spliced aligner with low memory requirements. *Nat Methods* 12:357-360.

Li, H., B. Handsaker, A. Wysoker, T. Fennell, J. Ruan, N. Homer, G. Marth, G. Abecasis, R. Durbin, and S. Genome Project Data Processing. 2009. The Sequence Alignment/Map format and SAMtools. *Bioinformatics* 25:2078-2079.

Quinlan, A.R., and I.M. Hall. 2010. BEDTools: a flexible suite of utilities for comparing genomic features. *Bioinformatics* 26:841-842.

Ramirez, F., D.P. Ryan, B. Gruning, V. Bhardwaj, F. Kilpert, A.S. Richter, S. Heyne, F. Dundar, and T. Manke. 2016. deepTools2: a next generation web server for deep-sequencing data analysis. *Nucleic acids research* 44:W160-165.

Rentas, S., N.T. Holzapfel, M.S. Belew, G.A. Pratt, V. Voisin, B.T. Wilhelm, G.D. Bader, G.W. Yeo, and K.J. Hope. 2016. Musashi-2 attenuates AHR signalling to expand human haematopoietic stem cells. *Nature* 532:508-511.

Schmittgen, T.D., and K.J. Livak. 2008. Analyzing real-time PCR data by the comparative C(T) method. *Nature protocols* 3:1101-1108.

Yu, G., L.G. Wang, Y. Han, and Q.Y. He. 2012. clusterProfiler: an R package for comparing biological themes among gene clusters. *OMICS* 16:284-287.

Yu, G., L.G. Wang, and Q.Y. He. 2015. ChIPseeker: an R/Bioconductor package for ChIP peak annotation, comparison and visualization. *Bioinformatics* 31:2382-2383.
